# Plasmonic photosynthesis of C_1_–C_3_ hydrocarbons from carbon dioxide assisted by an ionic liquid

**DOI:** 10.1038/s41467-019-10084-5

**Published:** 2019-05-01

**Authors:** Sungju Yu, Prashant K. Jain

**Affiliations:** 1 Department of Chemistry, University of Illinois at Urbana-Champaign, Urbana, Illinois 61801 USA; 2 Department of Physics, University of Illinois at Urbana-Champaign, Urbana, Illinois 61801 USA; 3 Materials Research Laboratory, University of Illinois at Urbana-Champaign, Urbana, Illinois 61801 USA; 4 Beckman Institute of Advanced Science and Technology, University of Illinois at Urbana-Champaign, Urbana, Illinois 61801 USA

**Keywords:** Photocatalysis, Energy, Nanoparticles

## Abstract

Photochemical conversion of CO_2_ into fuels has promise as a strategy for storage of intermittent solar energy in the form of chemical bonds. However, higher-energy-value hydrocarbons are rarely produced by this strategy, because of kinetic challenges. Here we demonstrate a strategy for green-light-driven synthesis of C_1_–C_3_ hydrocarbons from CO_2_ and H_2_O. In this approach, plasmonic excitation of Au nanoparticles produces a charge-rich environment at the nanoparticle/solution interface conducive for CO_2_ activation, while an ionic liquid stabilizes charged intermediates formed at this interface, facilitating multi-step reduction and C–C coupling. Methane, ethylene, acetylene, propane, and propene are photosynthesized with a C_2+_ selectivity of ~50% under the most optimal conditions. Hydrocarbon turnover exhibits a volcano relationship as a function of the ionic liquid concentration, the kinetic analysis of which coupled with density functional theory simulations provides mechanistic insights into the synergy between plasmonic excitation and the ionic liquid.

## Abstract (web summary)

While light-driven conversion of CO_2_ and H_2_O directly into fuels affords an attractive means to store sunlight in chemical bonds, few systems produce high-value hydrocarbons. Here, authors show gold nanoparticles to reduce CO_2_ to multi-carbon products using visible light, ionic liquids, and H_2_O.

## Introduction

Carbon dioxide (CO_2_) fixation is recognized to be a much-needed component of a carbon-neutral energy strategy^1–4^. Although CO_2_ is relatively unreactive, various catalytic processes triggered by heat (thermochemical)^5–8^, electricity (electrochemical)^9–17^, and light (photochemical)^18–28^ are being explored for activating CO_2_ and recycling it back to valuable petrochemicals. Sunlight-driven conversion of CO_2_ to fuels is particularly attractive as a means to store intermittent solar energy in the form of C–C and C–H bonds. Semiconductor and metal-catalyzed photoelectrolytic reduction of CO_2_ has shown promise; however, these processes have often required ultraviolet (UV) light and/or considerable electrical energy input, or they do not favor energy-rich hydrocarbon products. Longer-chain hydrocarbons possess higher energy densities. Moreover, hydrocarbons in the liquid state are easier to transport^29,30^. However, the formation of longer-chain hydrocarbons from CO_2_ requires multiple electron (e^–^) and proton (H^+^) transfer steps, as well as C–C bond formation^9,31,32^, which pose major kinetic bottlenecks.

**Fig. 1:**
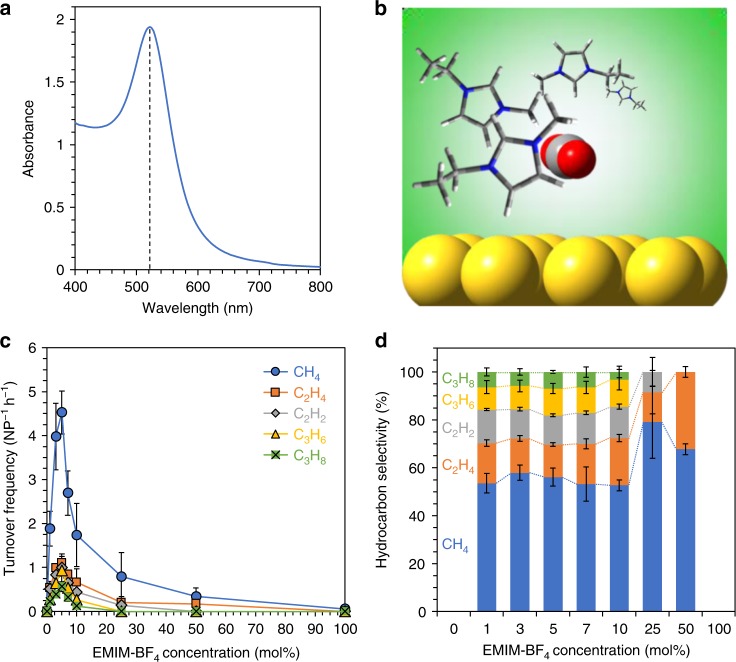
Ionic-liquid-promoted CO_2_ reduction to C_1_–C_3_ hydrocarbons using a plasmonic Au nanoparticle (NP) photocatalyst. **a** UV−vis extinction spectrum of a colloid of the Au NPs used for preparation of the photocatalyst film. The spectrum exhibits a localized surface plasmon resonance (LSPR) band centered around 520 nm, as indicated by the dotted line. **b** Scheme for CO_2_ conversion on plasmon-excited Au NPs promoted by an ionic liquid, EMIM-BF_4_. A continuous-wave (CW) laser of a wavelength of 532 nm and intensity of 1 W cm^–2^ was used as the light source for photoexcitation of Au NPs. EMIM-BF_4_ stabilizes CO_2_ and resulting adsorbates/intermediates on the photoexcited Au surface. **c** Turnover frequencies of hydrocarbon products formed in the CO_2_RR plotted as a function of the EMIM-BF_4_ concentration (mol%). The CO_2_ conversion activity peaks at 5 mol% of EMIM-BF_4_. **d** Hydrocarbon product selectivity as a function of EMIM-BF_4_ concentration (mol%). Each data point in **c** and **d** is the average of results from three identical trials and the error bar represents the SD of these measurements

Here we demonstrate a visible-light-driven route for the conversion of CO_2_ and H_2_O into C_1_–C_3_ hydrocarbons. The scheme does not involve the application of an electrochemical potential, UV light, high temperatures, hydrogen gas, or a sacrificial agent. It uses green light as the sole energy input and driving agent. The strategy employs plasmonic Au nanoparticles (NPs) of a pseudospherical shape and an average diameter of ~12 nm, as characterized previously^28^. Au NPs are known from electrochemical studies^33^ to activate CO_2_. The choice of Au NPs was further driven by the relative chemical stability of Au against bulk oxidation and photocorrosion; the other two common plasmonic metals, Ag and Cu, while electrocatalytically active for CO_2_ reduction, are prone to oxidation in air, water, and/or light excitation. The Au NPs possess a strong localized surface plasmon resonance (LSPR) band centered around 520 nm (Fig. 1a), which enables strong, resonant absorption of green light. The LSPR excitation of the NPs yields energetic electron–hole (e^–^–h^+^) carriers via Landau damping. These e^–^–h^+^ carriers were shown in recent studies to drive redox conversions^28,34–36^, especially the conversion of CO_2_ to methane and ethane under blue–green light^28^. However, in this past demonstration, isopropanol was used as a sacrificial h^+^ scavenger to facilitate e^–^–h^+^ pair separation; otherwise, unproductive e^–^–h^+^ recombination dominated. Thus, isopropanol served as the H^+^ source in this CO_2_ reduction scheme, which posed a major limitation for net energy storage.

The present strategy overcomes this drawback and uses water as the H^+^ source and does not require a sacrificial h^+^ scavenger, thus constituting a truly fuel-forming reaction. The enhanced reactivity was enabled by the use of an ionic liquid (IL) medium, specifically comprised 1-ethyl-3-methylimidazolium tetrafluoroborate (EMIM-BF_4_). Our choice was motivated by examples from electrocatalytic CO_2_ reduction reaction (CO_2_RR) where the EMIM-BF_4_ electrolyte, owing to its highly ionic character, stabilizes the high-energy CO_2_^•^^−^ radical anion intermediate formed in the reaction and decreases the overpotential needed for CO_2_RR^37–42^. In addition, EMIM-BF_4_ has a wide electrochemical window and high thermal stability^43,44^. In our photocatalytic scheme, the EMIM-BF_4_, as we find from kinetic analysis and density functional theory (DFT) simulations, promotes e^–^ transfer at the interface of the photoexcited Au NP and adsorbed CO_2_ (Fig. 1b), obviating the need for a h^+^ scavenger or applied potential for e^–^–h^+^ separation.

## Results

### IL-mediated plasmonic CO_2_ reduction

The photocatalyst had the form of a substrate-supported film of Au NPs immersed in an aqueous solution of EMIM-BF_4_ saturated with CO_2_ and contained inside a glass reactor (Supplementary Methods). The light excitation source comprised a continuous-wave (CW) laser of a wavelength of 532 nm light and an intensity of 1 W cm^−2^. Under CW excitation, the steady-state temperature of the reaction medium got moderately elevated to ~48 °C. Hydrocarbon products collected in the reactor headspace were measured (Supplementary Figs. 1−11) using a gas chromatograph (GC) equipped with a flame ionization detector. The EMIM-BF_4_ concentration was varied from 0 to 100 mol%, to find optimal conditions for CO_2_RR. In 1–10 mol% EMIM-BF_4_, the products of plasmon-excitation-driven CO_2_RR were found to be C_1_ (CH_4_), C_2_ (C_2_H_4_ and C_2_H_2_), and highly reduced C_3_ (C_3_H_6_ and C_3_H_8_) hydrocarbons (Fig. 1c, d and Supplementary Note 1). This product profile is quite striking when one considers that the major product in electrochemical CO_2_RR is carbon monoxide (CO) formed by 2e^–^–2H^+^ reduction of CO_2_ (refs. ^13–17^). On the other hand, propane (C_3_H_8_), formed in our scheme, requires an overall 20e^–^–20 H^+^ reduction and coupling of three CO_2_ molecules. Such generation of C_3_ hydrocarbons by artificial photosynthesis is challenging and therefore rare.

The CO_2_RR activity depends on the IL concentration (Fig. 1c). In pure water the activity was nil, whereas in 1 mol% EMIM-BF_4_ solution the generation of C_1_, C_2_, and C_3_ hydrocarbons was observed. The CO_2_RR activity, as quantified by turnover frequencies (TOFs) of the hydrocarbon products, increased dramatically with an increase in the EMIM-BF_4_ concentration. The highest activity was found at 5 mol% EMIM-BF_4_. Increasing the EMIM-BF_4_ concentration further resulted in a sharp drop in the CO_2_RR activity. In 100 mol% EMIM-BF_4_ solution, the activity was nil, similar to that in pure water. Thus, the CO_2_RR activity exhibits a volcano relationship as a function of the EMIM-BF_4_ concentration (Fig. 1c). At all EMIM-BF_4_ concentrations, where C_1_, C_2_, and C_3_ hydrocarbons were produced, the product selectivity was found to follow the order: C_1_ > C_2_ > C_3_. The selectivity for C_2+_ production is ~50% in 1−10 mol% EMIM-BF_4_ solution (Fig. 1d).

Non-hydrocarbon products were also characterized by a GC equipped with a thermal conductivity detector (TCD) (Supplementary Figs. 12–15). Considerable hydrogen (H_2_) production was measured (Supplementary Fig. 12), the TOF of which was 138.2 NP^−^^1^ h^−1^ in 5 mol% EMIM-BF_4_ solution, the IL concentration where CO_2_RR activity is the highest. The H_2_ likely originates from the competing reduction of H^+^ in the reaction medium (Supplementary Eq. (6)). In the GC-TCD measurements, there were no detection of CO (Supplementary Fig. 15), otherwise known to be a major product in electrocatalytic CO_2_RR on Au (refs. ^13–17^). Of the possible oxidation products, there was no measurable production of O_2_ (see Supplementary Information). H_2_O_2_ was detected (Supplementary Figs. 16–18) by the fluorogenic test employing a amplex red and horseradish peroxidase reagent^45^. Thus, the oxidation of H_2_O to H_2_O_2_ and H^+^ (2H_2_O → H_2_O_2_ + 2H^+^ + 2e^−^) is the likely oxidation half-reaction that consumes the photogenerated h^+^.

Control studies were performed, one without Au NPs, another without light, and a third without CO_2_. The conditions were otherwise maintained the same as those in the photoreaction tests and a 5 mol% EMIM-BF_4_ solution, found to be most optimal in the photoreaction tests, was employed. The control studies showed that the absence of any one of the components Au NPs, green light illumination, or CO_2_ resulted in nil hydrocarbon production, despite the use of 5 mol% EMIM-BF_4_ solution (Supplementary Fig. 19a–c). Thus, it is confirmed that the hydrocarbon production originates from green-light-driven CO_2_ reduction on Au NPs. The control study without light excitation was performed at an elevated temperature of 50 °C so as to mimic the steady-state bulk solution temperature of the reaction mixture in the photoreaction tests. The lack of CO_2_RR activity in this dark control study demonstrates that the CO_2_RR activity in the photoreaction tests does not originate from simply a photothermal effect of the light excitation. Rather a photoredox process facilitated by the Au NPs and the IL is responsible for the conversion of CO_2_ to hydrocarbons.

The plasmonic catalyst also exhibited stability and recyclability under the photoreaction conditions and IL media subjected on the catalyst. We tested the same substrate-supported Au NP film immersed in 5 mol% EMIM-BF_4_ over multiple cycles, each consisting of a 10 h photoreaction. The CO_2_RR activity and product selectivity, as determined from the TOFs of the hydrocarbon products, was maintained over the course of this multi-cycle test (Supplementary Fig. 20). As the NP film or EMIM-BF_4_ solution were not replenished between cycles, the maintenance of CO_2_RR activity over multiple cycles suggests that Au and EMIM-BF_4_ were not consumed, at any discernible levels, in the photoredox reaction.

### The origin of products

**Fig. 2:**
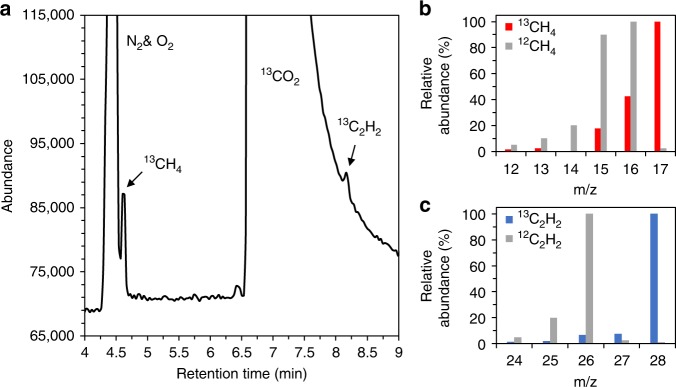
^13^CO_2_ isotopolog confirmation of CO_2_RR. **a** Total ion chromatogram (TIC) of the gaseous products from a 240 h long Au NP-photocatalyzed ^13^CO_2_RR in 5 mol% EMIM-BF_4_ solution under CW irradiation of 532 nm light (1 W cm^–2^). Peaks in the TIC appearing at retention times of 4.6 min and 8.2 min correspond to ^13^CH_4_ and ^13^C_2_H_2_. The basis for this assignment is provided in Supplementary Fig. 22. **b** Mass fragmentation pattern (red bars) acquired at a retention time of 4.6 min of the TIC shown in **a**. For comparison, a reference fragmentation pattern (gray bars) of ^12^CH_4_ from the National Institute of Standards and Technology (NIST) Chemistry WebBook is shown. Fragments at m/z = 14 and 18 in the experimental pattern were suppressed to remove the mass peaks contributed by N_2_ and moisture, respectively. **c** Mass fragmentation pattern (blue bars) acquired at a retention time of 8.2 min of the TIC shown in **a**. For comparison, a reference fragmentation pattern (gray bars) of ^12^C_2_H_2_ from the NIST Chemistry WebBook is shown. The fragment at m/z = 28 in the experimental pattern has relatively high abundance as compared with that of the reference fragmentation pattern due to the contribution of N_2_ from the atmosphere. Relative abundances in **b** and **c** were obtained from the measured abundances shown in Supplementary Fig. 21a, b, respectively. It is noteworthy that ^13^C_2_H_4_ was not resolved by GC-MS due to the likely overlap of the ^13^C_2_H_4_ peak with the broad, intense ^13^CO_2_ peak in the TIC (Supplementary Fig. 22b)

Given the hydrocarbon profile of the product mixture, it was necessary to go beyond the control studies described above and confirm more directly that CO_2_, rather than carbon contamination or photolysis of the EMIM-BF_4_, was the source of the hydrocarbon products. For this confirmation, ^13^C isotope labeling was employed (Fig. 2 and Supplementary Figs. 21 and 22). In this labeling study, ^13^CO_2_ was employed as the reactant instead of ^12^CO_2_, whereas all other conditions were kept the same as those in other photoreaction tests. GC-mass spectrometry (GC-MS) was used for identification of the hydrocarbon products generated in the photoreaction (Fig. 2a). The GC-MS analysis confirmed the presence of ^13^CH_4_ (Fig. 2b) and ^13^C_2_H_2_ (Fig. 2c), manifested by their characteristic mass fragmentation patterns, shifted to higher m/z compared with reference fragmentation patterns of ^12^CH_4_ and ^12^C_2_H_2_, respectively. Thus, isotope labeling confirms CO_2_ to be the origin of hydrocarbon products.

### The role of the IL

We attempted to gain a mechanistic understanding of this catalytic scheme focusing on the question of how the IL promotes CO_2_RR activity. It was observed that the presence of EMIM-BF_4_ in the aqueous medium results in a considerably acidic pH (Supplementary Fig. 23): the 5 mol% EMIM-BF_4_ solution has a pH of 2.95. To determine whether this acidity is responsible for the enhanced CO_2_RR activity in a EMIM-BF_4_ solution, we performed a photoreaction in deionized water containing no EMIM-BF_4_ but with a pH of 2.93 achieved using acid (Supplementary Fig. 19d). All other conditions were kept the same as in the photoreactions in EMIM-BF_4_ solutions. In this EMIM-BF_4_-free photoreaction, no products were observed, which demonstrated that the high acidity or H^+^ concentration, [H^+^], of the EMIM-BF_4_-containing medium is not the sole cause of the enhanced CO_2_RR activity. EMIM-BF_4_ plays other role(s). It is possible, in principle, for EMIM-BF_4_, instead of H_2_O, to serve as the h^+^ acceptor; however, if this were the case, then the CO_2_RR activity would have been enhanced at higher EMIM-BF_4_ concentrations, in line with a study of a different plasmon excitation-catalyzed redox reaction^36^. Instead, we observed peak activity at a EMIM-BF_4_ concentration of 5 mol%, above which the activity drops steeply reaching nil in pure EMIM-BF_4_ wherein H_2_O is not available.

**Fig. 3:**
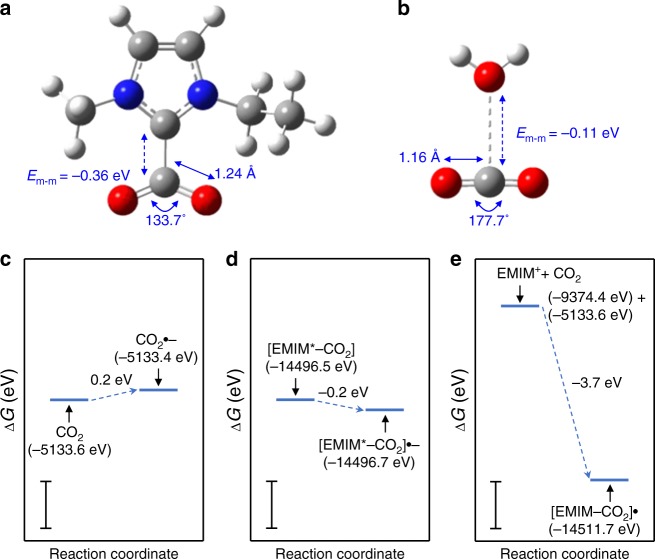
The role of the ionic liquid in Au NP-photocatalyzed CO_2_RR. **a**,**b** DFT-optimized geometries of [EMIM*-CO_2_] (**a**) and [H_2_O-CO_2_] (**b**) complexes. C, H, O, and N atoms are depicted by gray, white, red, and blue spheres, respectively. Key bond lengths, bond angles, and the energy of intermolecular interaction, *E*_m-m_, are indicated for each of the complexes. **c**–**e** DFT-computed free energy cost, ∆*G*, of formation of the 1e^−^ adduct of CO_2_ (**c**), 1e^−^ adduct of [EMIM*-CO_2_] (**d**), and 1e^−^ adduct of CO_2_ in the presence of EMIM^+^ (**e**). In the latter case, the 1e^−^ adduct of CO_2_, CO_2_^•−^, is stabilized by complexation with EMIM^+^ as described by the net process: EMIM^+^ + CO_2 _+ e^−^→ [EMIM-CO_2_]^•^. In **c**–**e**, the free energy of each species is indicated in parentheses. Scale bars are 1 eV in length

We hypothesized that the strongly ionic character of EMIM-BF_4_ plays a role in the activation of CO_2_, which is otherwise fairly redox inactive. CO_2_, however, is highly polarizable, as indicated by its quadrupole moment of −4.3 D Å (ref. ^46^). The interaction of EMIM-BF_4_ and CO_2_ was simulated by DFT. A past study suggests that CO_2_ can undergo complexation with the *N*-heterocyclic carbene, EMIM^*^, formed from EMIM^+^ by H^+^ loss^42^. We investigated using DFT the structure of such a [EMIM*-CO_2_] complex (Fig. 3a). The complex exhibits binding between the C atom of the CO_2_ and the C_2_ atom of the imidazole ring with an energy of intermolecular interaction, *E*_m-m_, of −0.36 eV. This interaction is stronger than, for instance, the interaction of an H_2_O molecule and CO_2_ (Fig. 3b). Unlike the latter case, complexation with EMIM^*^ leads to considerable restructuring of the CO_2_ moiety. The CO_2_ moiety adopts a bent configuration with an O=C=O angle of 133.7° and C=O bonds lengthened to 1.24 Å. In fact, the geometry of the CO_2_ moiety in the complex closely mirrors that of the CO_2_^•−^ anion radical, which has a bond angle of 137.8° and bond length of 1.23 Å (Supplementary Fig. 24). Moreover, from Mulliken charge partitioning analysis (Supplementary Fig. 25), the CO_2_ moiety in the [EMIM*-CO_2_] complex is found to have a net charge of −0.73, which indicates its partial anionic character.

It is known that the energetic cost of the drastic structural reorganization from linear CO_2_ to the bent CO_2_^•−^ anion radical poses a major barrier for e^−^ acceptance by CO_2_ (refs. ^37–42^). However, our DFT calculations show that in its complex with EMIM*, the CO_2_ moiety is structurally pre-configured for e^−^ acceptance. Consistent with this finding, 1e^–^ addition to [EMIM*-CO_2_] is much more favorable as compared with 1e^−^ addition to CO_2_ (Fig. 3c, d). Thus, it appears that EMIM-BF_4_ can promote the transfer of photogenerated e^–^ from the Au NP to adsorbed CO_2_, which is otherwise a major kinetic bottleneck in the photocatalytic reduction process. Furthermore, it is plausible that the CO_2_^•−^ anion radical formed on the Au surface by photo-initiated e^–^ transfer process has an enhanced lifetime due to solvation or complexation by EMIM^+^ (Fig. 3e). A longer lifetime of this reactive intermediate would increase the probability of C–C coupling between the intermediates.

### Empirical kinetic model

Although the DFT computations provide insight into the central role of EMIM-BF_4_ in CO_2_ activation, the volcano-type dependence of the CO_2_RR activity on the IL concentration deserves an explanation. From the hydrolysis of EMIM-BF_4_ known from past studies^47–50^:

1 $${\mathrm{EMIM-BF}}_4 + x{\mathrm{H}}_2{\mathrm{O}} \to {\mathrm{EMIM}}^ + + \left[ {{\mathrm{BF}}_{4-x}\left( {{\mathrm{OH}}} \right)_x} \right]^- + x{\mathrm{HF}}$$

where *x* = 1–4 and the complexation of CO_2_ with EMIM^+^ predicted in DFT simulations:

2 $${\mathrm{EMIM}}^ + + {\mathrm{CO}}_2 \to \left[ {{\mathrm{EMIM}}^ \ast{\mathrm{-CO}}_2} \right] + {\mathrm{H}}^ +$$

we postulate a rate determining step in the reaction of CO_2_ and H_2_O:

3 $${\mathrm{EMIM-BF}}_4 + {\mathrm{CO}}_2 + x{\mathrm{H}}_2{\mathrm{O}} \to \left[ {{\mathrm{EMIM}}^\ast{\mathrm{-CO}}_2} \right] \\ + \left[ {{\mathrm{BF}}_{4-x}\left( {{\mathrm{OH}}} \right)_x} \right]^- + \left( {x + 1} \right){\mathrm{H}}^ + + x{\mathrm{F}}^-$$

From this reaction equation, the concentration of the activated CO_2_ complex, [EMIM*-CO_2_], is expected to be directly proportional to [H^+^]^*x*+1^. Therefore, the [H^+^] determined from the measured pH of the EMIM-BF_4_ solution (Supplementary Fig. 23) serves as a proxy for the concentration of [EMIM*-CO_2_], based on which the [EMIM*-CO_2_] concentration is expected to be the highest in the EMIM-BF_4_ concentration range around 5 mol%. The higher the concentration of the activated [EMIM*-CO_2_] complex, the greater is the rate of CO_2_ conversion and also the higher the likelihood of C–C coupling required for C_2+_ production. Therefore, both the overall activity and the selectivity in favor of C_2+_ products are favorable in the 3–7 mol% EMIM-BF_4_ range, with the most optimal performance achieved at 5 mol% EMIM-BF_4_. On the other hand, the activated complex has zero concentration in pure water on one extreme and in pure EMIM-BF_4_ on the other extreme, which explains the nil turnover at these conditions. An additional reason for the drop in activity at higher EMIM-BF_4_ concentrations may be that the adsorption of BF_4_^–^ to the Au NP surface (Supplementary Fig. 26) dominates at these concentrations to such an extent that the adsorption of CO_2_ and/or [EMIM*-CO_2_] to the Au surface is largely inhibited and so is the e^−^ transfer to CO_2_.

**Fig. 4:**
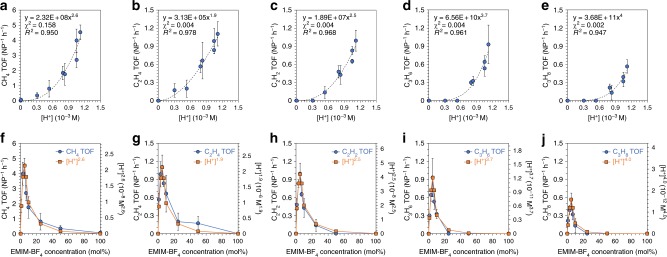
Empirical kinetic model for the CO_2_RR. **a**–**e** For each of the hydrocarbon products, the TOF is plotted as a function of the H^+^ concentration, [H^+^], which serves as a proxy for the concentration of the [EMIM*-CO_2_] complex. A fit to a power-law function *y* = *ax*^*n*^ (dashed line) yields the fit parameter *n*, which represents the apparent reaction order in [H^+^]. The best-fit equation is indicated for each plot along with the values of *χ*^2^ and *R*^2^, which serve as metrics of the goodness-of-fit. **f**–**j** For each hydrocarbon product, the TOF plotted as a function of the EMIM-BF_4_ concentration follows a similar trend as the [H^+^]^*n*^, where *n* is the corresponding reaction order obtained from the plots in **a**–**e**. Each data point in **a**–**j** is the average of results from three identical trials and the error bar represents the SD of these measurements

The CO_2_RR activity depends on the concentration of this activated complex to a high reaction order. This is best exemplified by the plots of TOF for each hydrocarbon as a function of the [H^+^] (Fig. 4a–e), which as explained above, serves as a proxy for the concentration of [EMIM*-CO_2_]. The pseudo-reaction order, *n*, is found to be 1.9 for C_2_H_4_, 2.5 for C_2_H_2_, 3.7 for C_3_H_6_, and 4.0 for C_3_H_8_. The fit for the CH_4_ TOF has a relatively high *χ*^2^-value, so the *n* of 2.7 estimated for CH_4_ has a lower confidence. In general, the pseudo-reaction order is higher for the longer hydrocarbons, which perhaps captures the need for multiple activated complexes to be available for undergoing coupling to C_2_ and C_3_ fragments. The high pseudo-reaction order for the C_3_ products goes hand-in-hand with an apparent threshold in [H^+^] below which the TOF is zero or below the detection limit (Fig. 4d, e). For each of the hydrocarbon products, the [H^+^] raised to the power of the corresponding *n* follows a volcano trend with respect to the EMIM-BF_4_ concentration, mirroring closely the trend in the TOF for that hydrocarbon (Fig. 4f–j).

Thus, we reported the green-light-driven synthesis of C_1_–C_3_ hydrocarbons from CO_2_ and water on plasmonic Au NPs in an IL medium. The resonant green light absorption of the plasmonic NPs and their ability to sustain electrostatically charged surfaces under resonant CW excitation are at the heart of the observed photoreactivity. The IL plays a synergistic role due to its complexation with the CO_2_, which preconfigures the CO_2_ for accepting e^–^ from photoexcited Au NPs. The enhanced reactivity of CO_2_ in the presence of the IL obviates the need for an applied potential or a sacrificial scavenger. Although hydrocarbon production yields in the reaction need further optimization, the generation of propane by overall 20e^−^–20H^+^ reduction and coupling of three CO_2_ molecules is both striking and mechanistically rich. The precise intermediates and reaction pathways, including C–C coupling and dehydrogenation steps, which yield each of the hydrocarbons, deserve further elucidation. Beyond CO_2_ conversion studied here, ILs may have promise in other photocatalytic schemes where activation of relatively inert substrates and stabilization of high-energy charged intermediates is desirable.

## Supplementary information

Supplementary Information

## Data Availability

All raw images and source data are available from the authors upon reasonable request.
