# Supplementary material for: Plasmonic photosynthesis of C1–C3 hydrocarbons from carbon dioxide assisted by an ionic liquid
Source: Nat Commun. 2019 May 1;10:2022. doi: 10.1038/s41467-019-10084-5 (PMC6494896; doi:10.1038/s41467-019-10084-5)
Supplement: Supplementary file 1 — Supplementary Information [file 41467_2019_10084_MOESM1_ESM.pdf]

Supplementary Information

Plasmonic photosynthesis of C_1–C_3 hydrocarbons from carbon dioxide assisted by an ionic liquid

Yu and Jain

Supplementary Figures

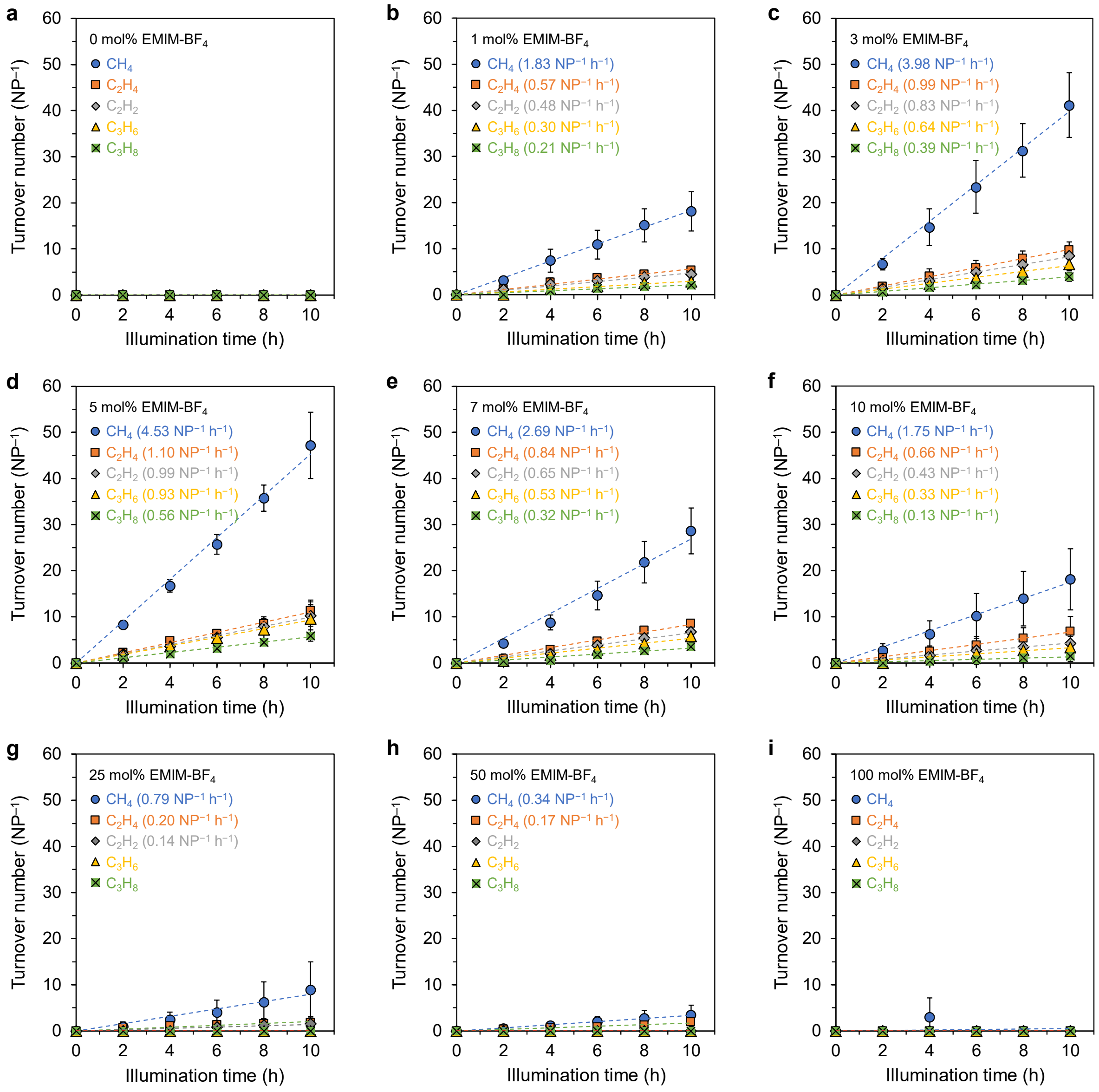

Supplementary Fig. 1 | Time course of hydrocarbon (CH_4, C_2H_4, C_2H_2, C_3H_6, and C_3H_8) production in CO_2 reduction reactions photocatalyzed by Au NPs with (a) 0 mol%, (b) 1 mol%, (c) 3 mol%, (d) 5 mol%, (e) 7 mol%, (f) 10 mol%, (g) 25 mol%, (h) 50 mol%, and (i) 100 mol% EMIM-BF_4 solutions. A CW 532 nm laser with an intensity of 1 W cm^{-2} was used as the light source. Each data point is the average of results from three identical trials, and the error bar represents the standard deviation of these measurements. Each dashed line is a linear fit to the plots of TON for each product vs. illumination time. The slope yields the average TOF of each product, which is noted with the corresponding product in each plot. These TOF values are plotted in Fig. 1c and Fig. 4. For allowing appropriate comparison, all the plots have the same y-axis scale as the plot in panel (d), which represents the EMIM-BF_4 concentration at which the highest activity was measured.

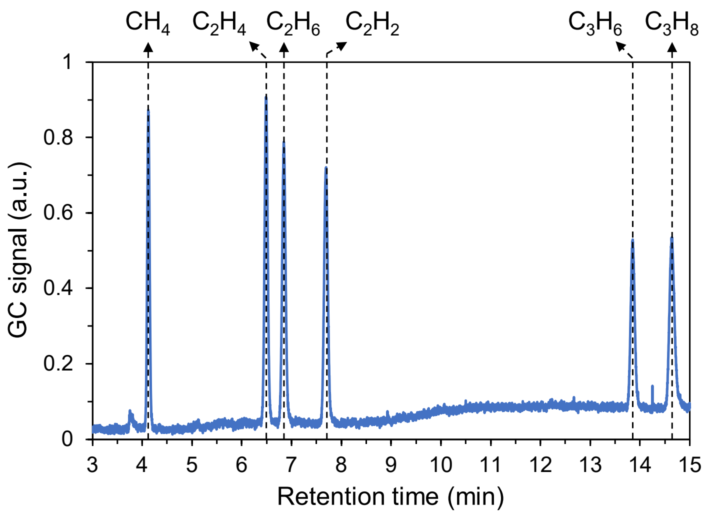

Supplementary Fig. 2 | Reference GC-FID chromatogram of a calibration gas mixture of CH_4, C_2H_4, C_2H_6, C_2H_2, C_3H_6, and C_3H_8. GC peaks for hydrocarbons products (Supplementary Figs. 3−11) were assigned by comparison with this reference chromatogram.

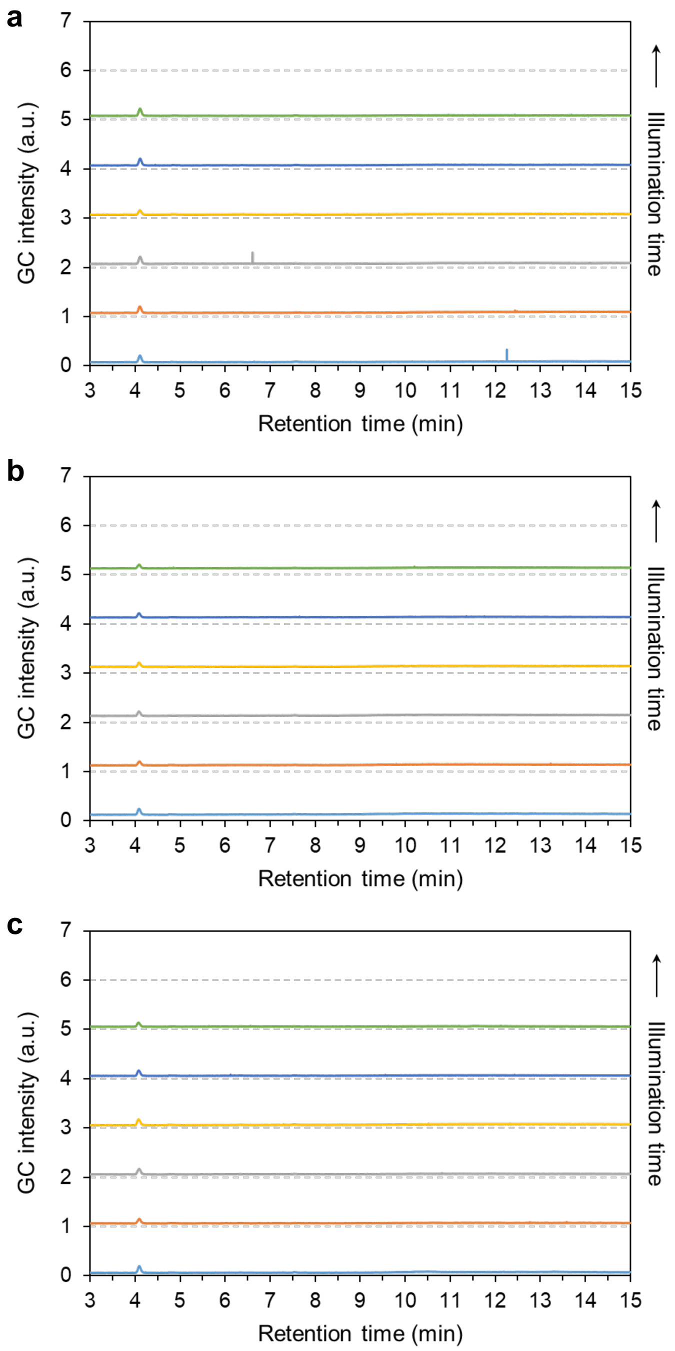

Supplementary Fig. 3 | (a–c) GC-FID chromatograms obtained at 2 h intervals in a 10 h long Au-photocatalyzed CO_2 reduction reaction with 0 mol% EMIM-BF_4 and CW irradiation of 532 nm light (1 W cm^{-2}). Three identical trials were performed, the results from which are shown in the three panels above. Chromatograms are plotted vertically stacked from the bottom to top in order of illumination time: 0 h, 2 h, 4 h, 6 h, 8 h, and 10 h. The small GC peak observed near 4.1 min is from CH_4 as indicated by a comparison with the reference chromatogram in Supplementary Fig. 2. Thus, the chromatograms only show a trace of CH_4 as an impurity originating from CO_2 gas. No significant generation of hydrocarbon products was observed under these photoreaction conditions. The TON vs. illumination time plot resulting from the analysis of these chromatograms is shown in Supplementary Fig. 1a.

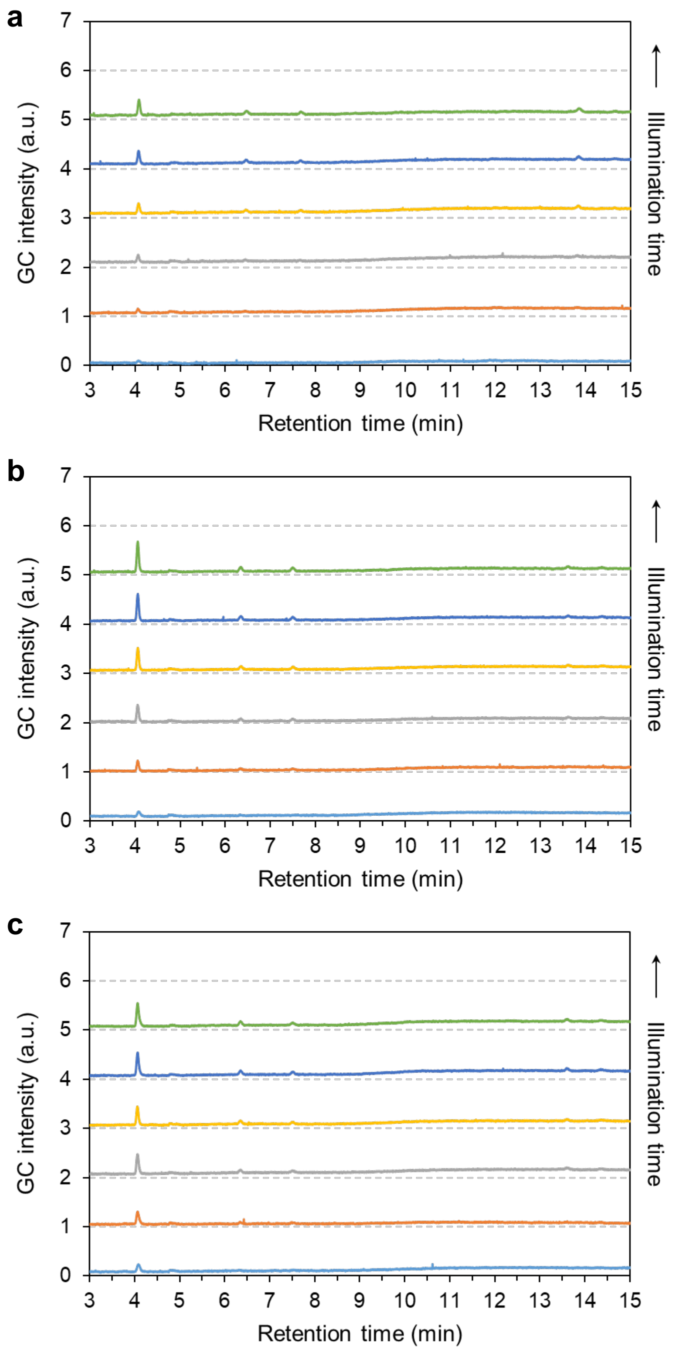

Supplementary Fig. 4 | (a–c) GC-FID chromatograms obtained at 2 h intervals in a 10 h long Au-photocatalyzed CO_2 reduction reaction with 1 mol% EMIM-BF_4 and CW irradiation of 532 nm light (1 W cm^{-2}). Three identical trials were performed, the results from which are shown in the three panels above. Chromatograms are plotted vertically stacked from the bottom to top in order of illumination time: 0 h, 2 h, 4 h, 6 h, 8 h, and 10 h. The chromatograms show an increase in the intensity of GC peaks associated with CH_4, C_2H_4, C_2H_2, C_3H_6, and C_3H_8 with increasing illumination time. These peaks were assigned to hydrocarbon products by comparison with the reference chromatogram in Supplementary Fig. 2. The TON vs. illumination time plot resulting from the analysis of these chromatograms is shown in Supplementary Fig. 1b.

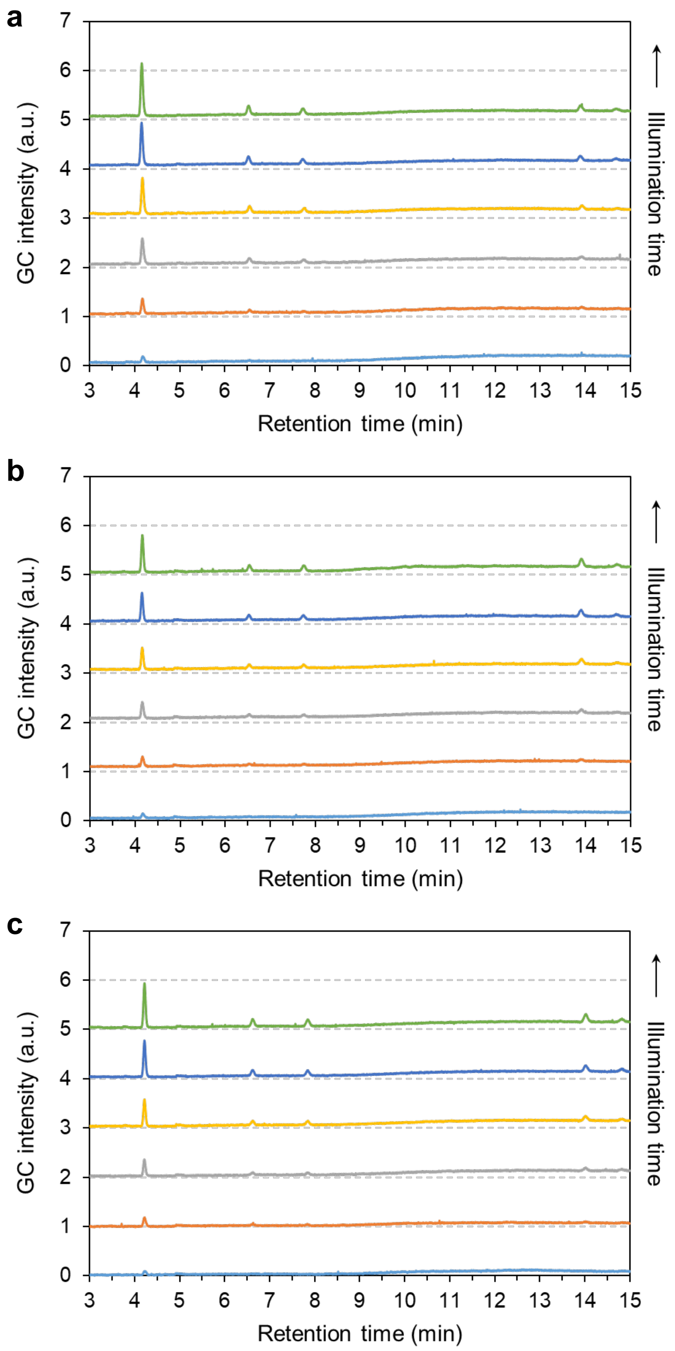

Supplementary Fig. 5 | (a–c) GC-FID chromatograms obtained at 2 h intervals in a 10 h long Au-photocatalyzed CO_2 reduction reaction with 3 mol% EMIM-BF_4 and CW irradiation of 532 nm light (1 W cm^{-2}). Three identical trials were performed, the results from which are shown in the three panels above. Chromatograms are plotted vertically stacked from the bottom to top in order of illumination time: 0 h, 2 h, 4 h, 6 h, 8 h, and 10 h. The chromatograms show an increase in the intensity of GC peaks associated with CH_4, C_2H_4, C_2H_2, C_3H_6, and C_3H_8 with increasing illumination time. These peaks were assigned to hydrocarbon products by comparison with the reference chromatogram in Supplementary Fig. 2. The TON vs. illumination time plot resulting from the analysis of these chromatograms is shown in Supplementary Fig. 1c.

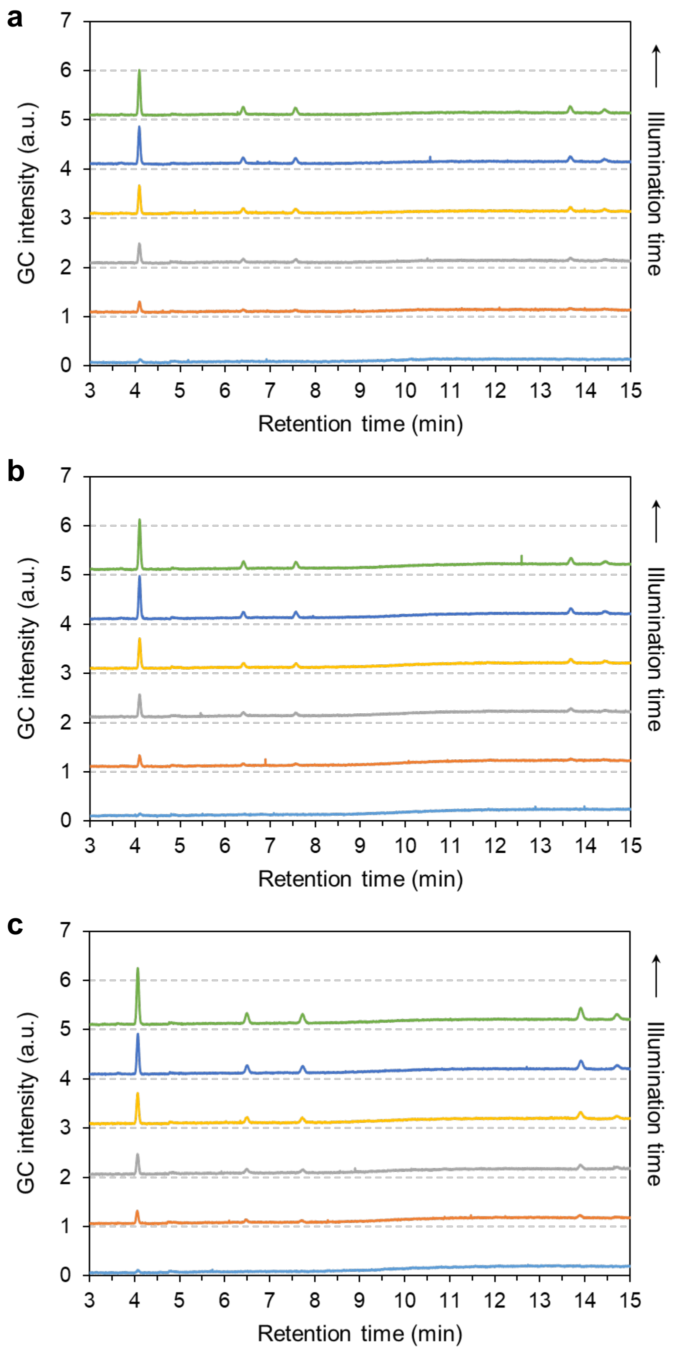

Supplementary Fig. 6 | (a–c) GC-FID chromatograms obtained at 2 h intervals in a 10 h long Au-photocatalyzed CO_2 reduction reaction with 5 mol% EMIM-BF_4 and CW irradiation of 532 nm light (1 W cm^{-2}). Three identical trials were performed, the results from which are shown in the three panels above. Chromatograms are plotted vertically stacked from the bottom to top in order of illumination time: 0 h, 2 h, 4 h, 6 h, 8 h, and 10 h. The chromatograms show an increase in the intensity of GC peaks associated with CH_4, C_2H_4, C_2H_2, C_3H_6, and C_3H_8 with increasing illumination time. These peaks were assigned to hydrocarbon products by comparison with the reference chromatogram in Supplementary Fig. 2. The TON vs. illumination time plot resulting from the analysis of these chromatograms is shown in Supplementary Fig. 1d.

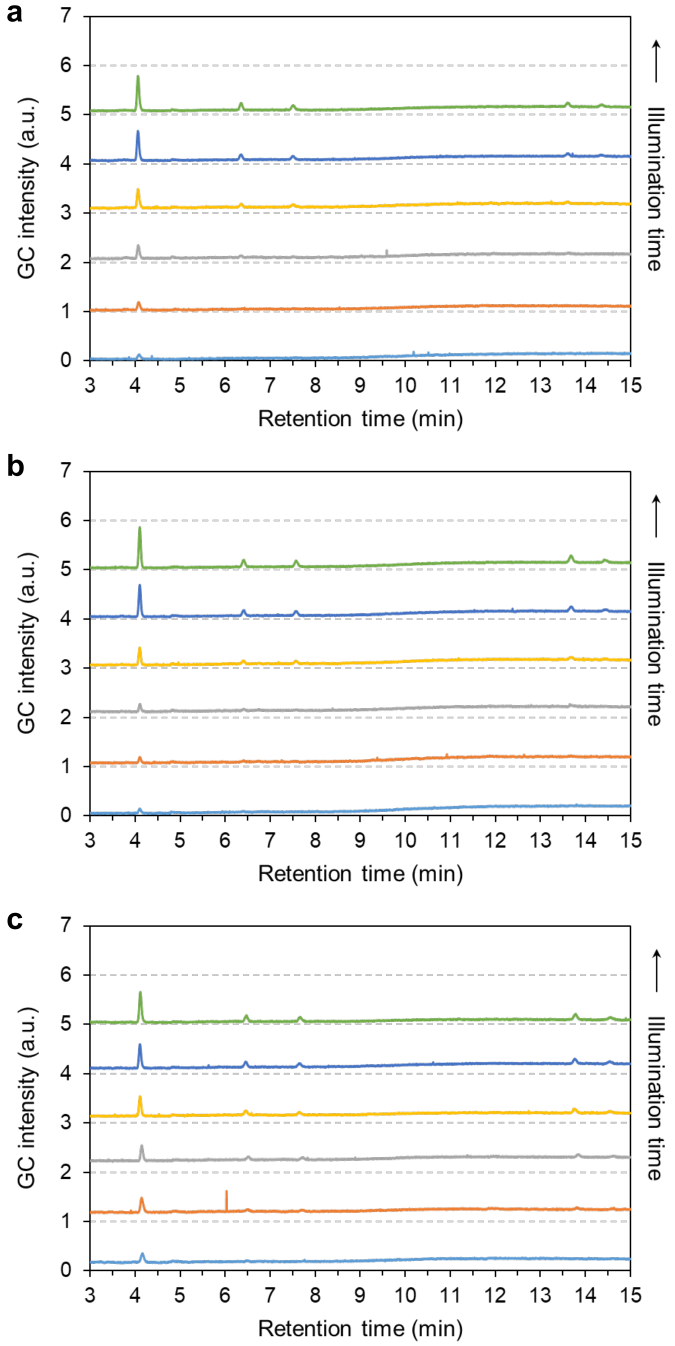

Supplementary Fig. 7 | (a–c) GC-FID chromatograms obtained at 2 h intervals in a 10 h long Au-photocatalyzed CO_2 reduction reaction with 7 mol% EMIM-BF_4 and CW irradiation of 532 nm light (1 W cm^{-2}). Three identical trials were performed, the results from which are shown in the three panels above. Chromatograms are plotted vertically stacked from the bottom to top in order of illumination time: 0 h, 2 h, 4 h, 6 h, 8 h, and 10 h. The chromatograms show an increase in the intensity of GC peaks associated with CH_4, C_2H_4, C_2H_2, C_3H_6, and C_3H_8 with increasing illumination time. These peaks were assigned to hydrocarbon products by comparison with the reference chromatogram in Supplementary Fig. 2. The TON vs. illumination time plot resulting from the analysis of these chromatograms is shown in Supplementary Fig. 1e.

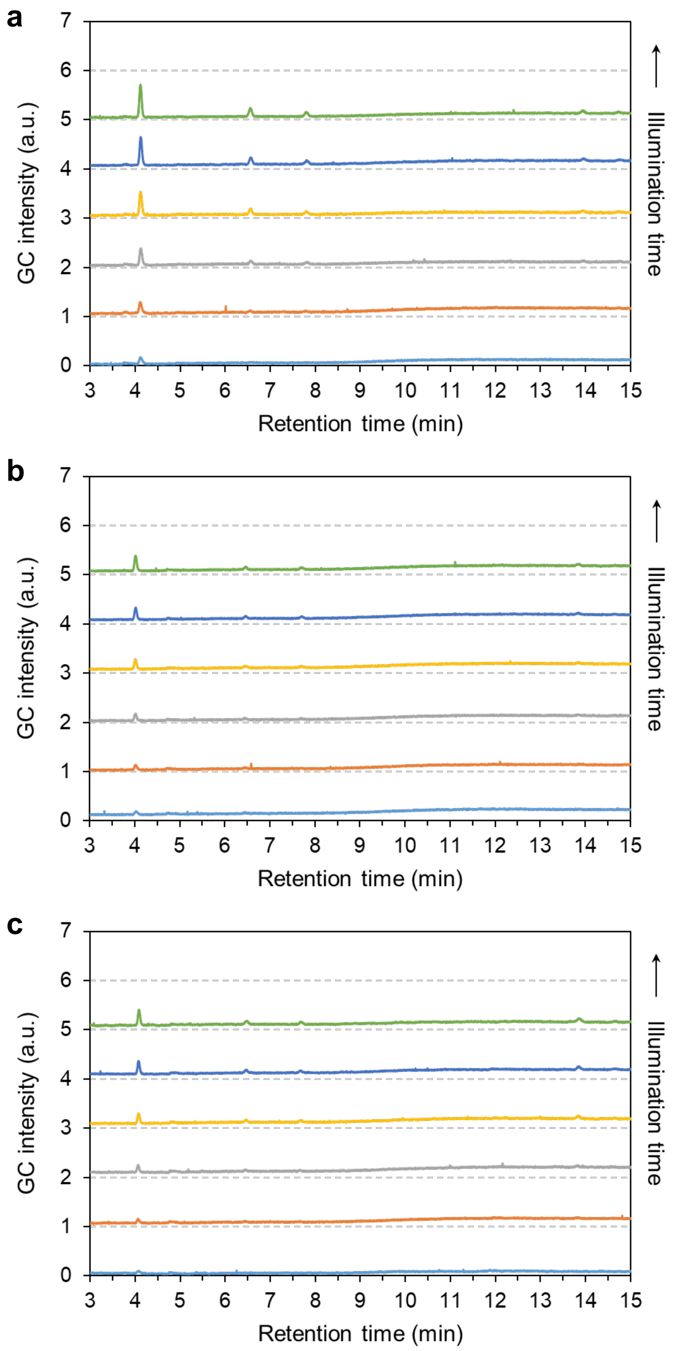

Supplementary Fig. 8 | (a–c) GC-FID chromatograms obtained at 2 h intervals in a 10 h long Au-photocatalyzed CO_2 reduction reaction with 10 mol% EMIM-BF_4 and CW irradiation of 532 nm light (1 W cm^{-2}). Three identical trials were performed, the results from which are shown in the three panels above. Chromatograms are plotted vertically stacked from the bottom to top in order of illumination time: 0 h, 2 h, 4 h, 6 h, 8 h, and 10 h. The chromatograms show an increase in the intensity of GC peaks associated with CH_4, C_2H_4, C_2H_2, C_3H_6, and C_3H_8 with increasing illumination time. These peaks were assigned to hydrocarbon products by comparison with the reference chromatogram in Supplementary Fig. 2. The TON vs. illumination time plot resulting from the analysis of these chromatograms is shown in Supplementary Fig. 1f.

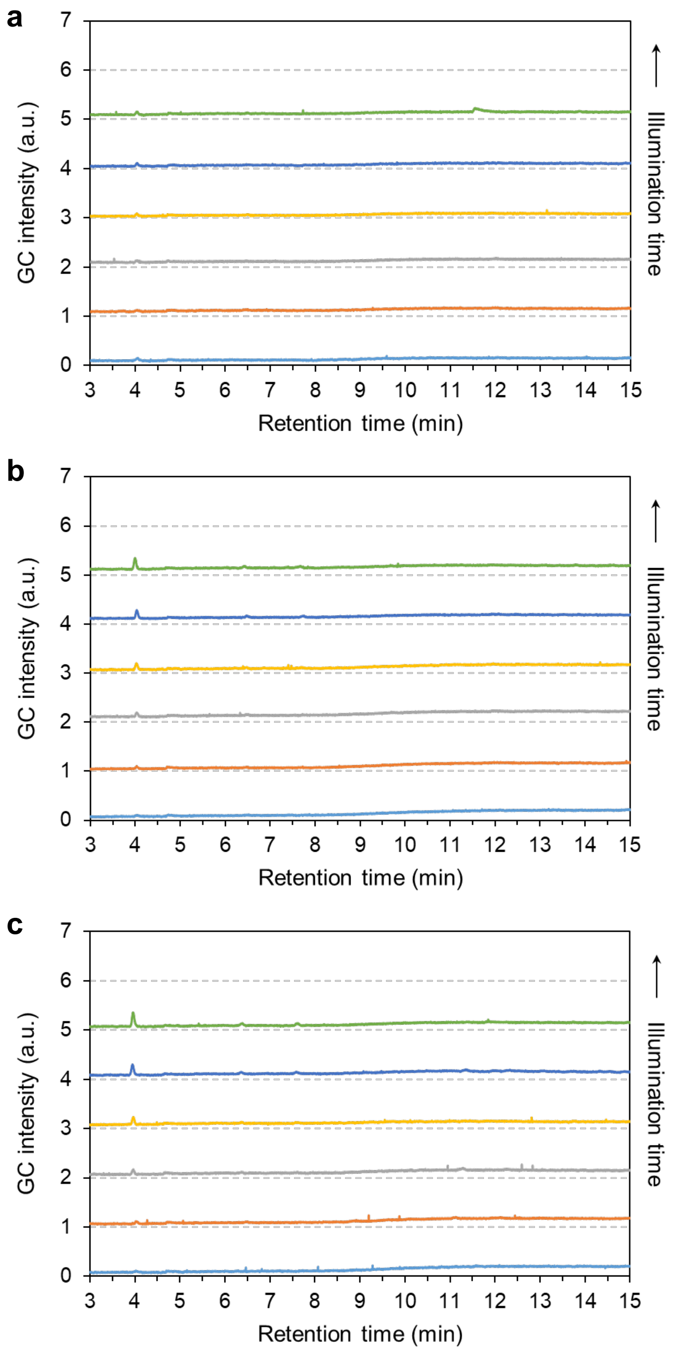

Supplementary Fig. 9 | (a–c) GC-FID chromatograms obtained at 2 h intervals in a 10 h long Au-photocatalyzed CO_2 reduction reaction with 25 mol% EMIM-BF_4 and CW irradiation of 532 nm light (1 W cm^{-2}). Three identical trials were performed, the results from which are shown in the three panels above. Chromatograms are plotted vertically stacked from the bottom to top in order of illumination time: 0 h, 2 h, 4 h, 6 h, 8 h, and 10 h. The chromatograms show an increase in the intensity of GC peaks associated with CH_4, C_2H_4, and C_2H_2 with increasing illumination time. These peaks were assigned to hydrocarbon products by comparison with the reference chromatogram in Supplementary Fig. 2. The TON vs. illumination time plot resulting from the analysis of these chromatograms is shown in Supplementary Fig. 1g.

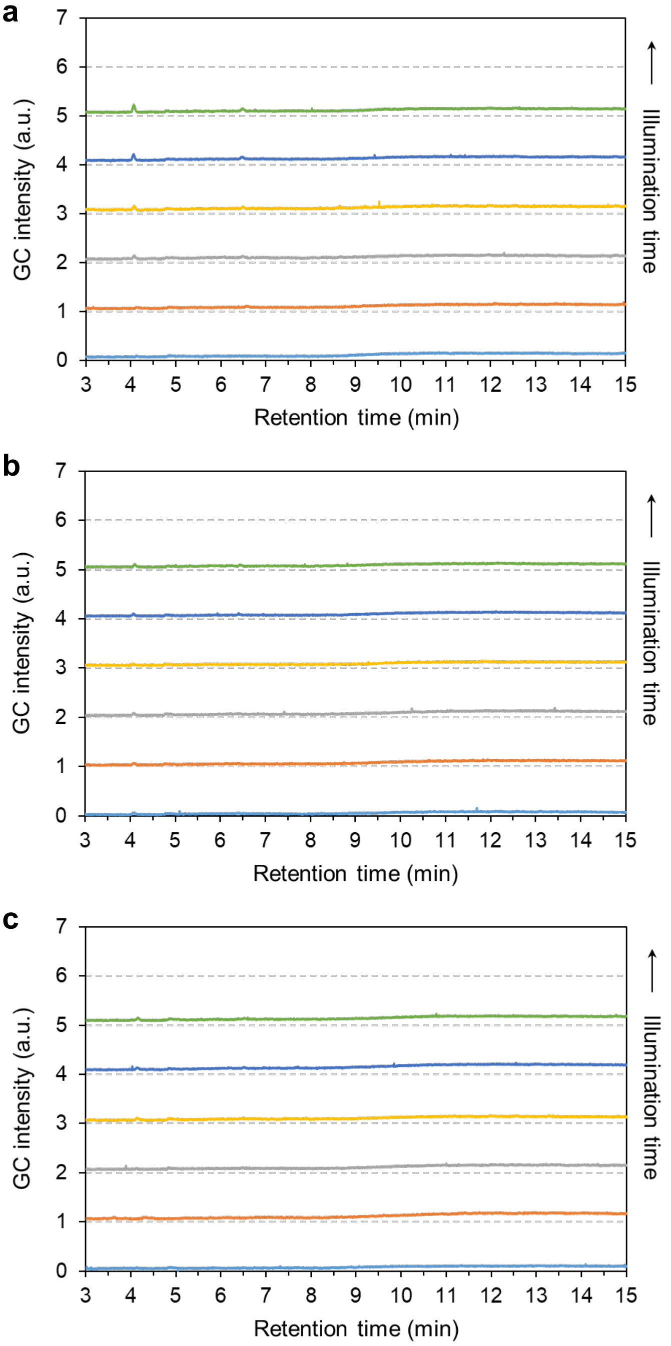

Supplementary Fig. 10 | (a–c) GC-FID chromatograms obtained at 2 h intervals in a 10 h long Au-photocatalyzed CO_2 reduction reaction with 50 mol% EMIM-BF_4 and CW irradiation of 532 nm light (1 W cm^{-2}). Three identical trials were performed, the results from which are shown in the three panels above. Chromatograms are plotted vertically stacked from the bottom to top in order of illumination time: 0 h, 2 h, 4 h, 6 h, 8 h, and 10 h. The chromatograms show an increase in the intensity of GC peaks associated with CH_4 and C_2H_4 with increasing illumination time. These peaks were assigned to hydrocarbon products by comparison with the reference chromatogram in Supplementary Fig. 2. The TON vs. illumination time plot resulting from the analysis of these chromatograms is shown in Supplementary Fig. 1h.

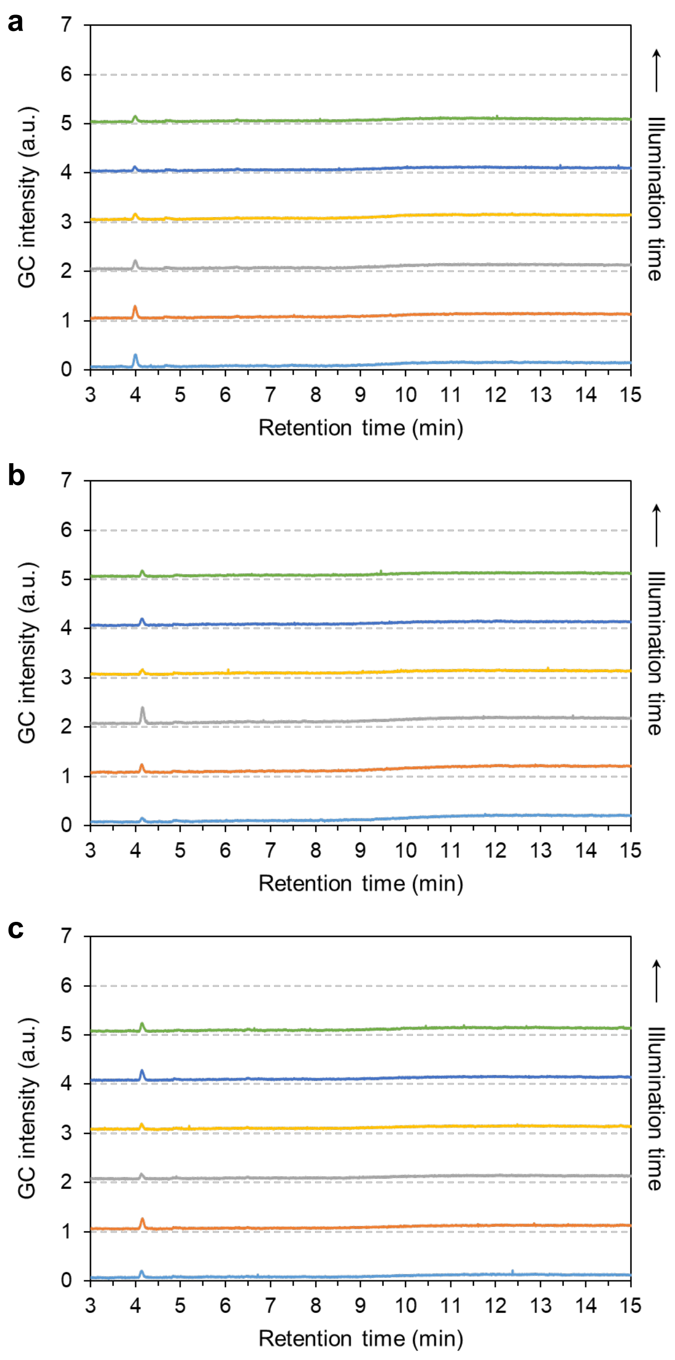

Supplementary Fig. 11 | (a–c) GC-FID chromatograms obtained at 2 h intervals in a 10 h long Au-photocatalyzed CO_2 reduction reaction with 100 mol% EMIM-BF_4 and CW irradiation of 532 nm light (1 W cm^{-2}). Three identical trials were performed, the results from which are shown in the three panels above. Chromatograms are plotted vertically stacked from the bottom to top in order of illumination time: 0 h, 2 h, 4 h, 6 h, 8 h, and 10 h. The small GC peak observed near 4.1 min is from CH_4 as indicated by a comparison with the reference chromatogram in Supplementary Fig. 2. Thus, the chromatograms only show a trace of CH_4 as an impurity originating from CO_2 gas. No significant generation of hydrocarbon products was observed under these photoreaction conditions. The TON vs. illumination time plot resulting from the analysis of these chromatograms is shown in Supplementary Fig. 1i.

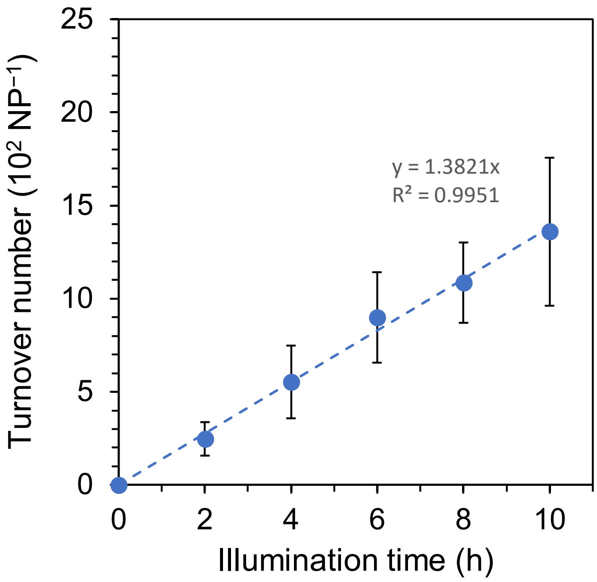

Supplementary Fig. 12 | Time course of H_2 turnover in the Au-photocatalyzed CO_2 reduction reaction with 5 mol% EMIM-BF_4 under 1 W cm^{-2}, 532 nm light. H_2 TOF is found to be 138.2 NP^{-1} h^{-1} from the slope of a linear fit (dashed line) to the plot of the H_2 TON vs. illumination time. Data points plotted are averages of measurements from three identical trials of the photoreaction. The standard deviation of the measurements is represented by the error bar.

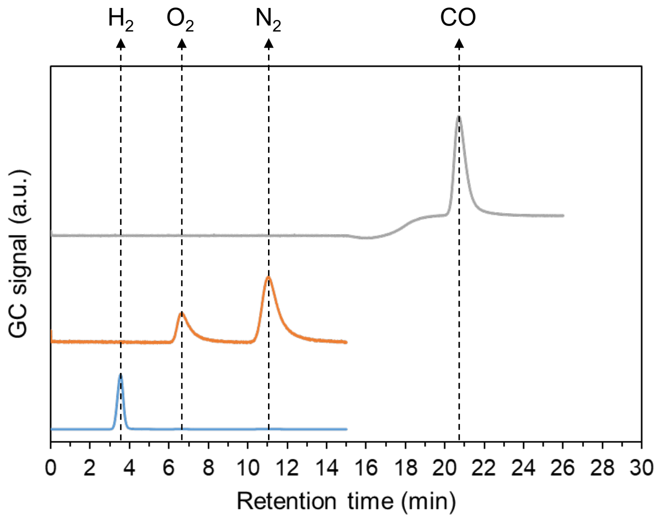

Supplementary Fig. 13 | Reference GC-TCD chromatograms of H_2 (blue curve), air (orange curve), and CO (gray curve) shown vertically stacked. GC peaks observed in chromatograms presented in Supplementary Figs. 14 and 15 were assigned by comparison with these reference chromatograms.

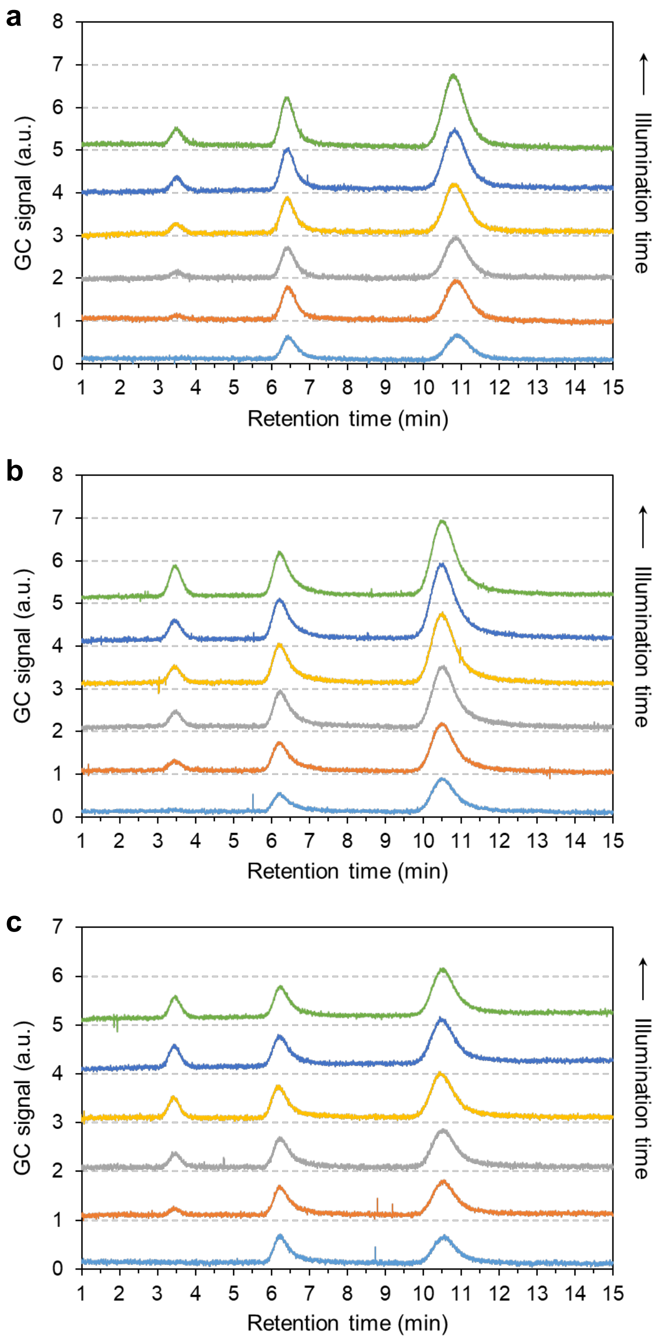

Supplementary Fig. 14 | Detection of non-hydrocarbon products using GC-TCD. (a–c) GC-TCD chromatograms obtained at 2 h intervals in a 10 h long Au-photocatalyzed CO_2 reduction reaction with 5 mol% EMIM-BF_4 and CW irradiation of 532 nm light (1 W cm^{-2}). Three identical trials were performed, the results from which are shown in the three panels above. Chromatograms are plotted vertically stacked from the bottom to top in order of illumination time: 0 h, 2 h, 4 h, 6 h, 8 h, and 10 h. The chromatograms show an increase in the intensity of GC peak associated with H_2 with increasing illumination time. The GC peaks were assigned to by comparison with the reference chromatogram in Supplementary Fig. 13. The TON vs. illumination time plot resulting from the analysis of these chromatograms is shown in Supplementary Fig. 12.

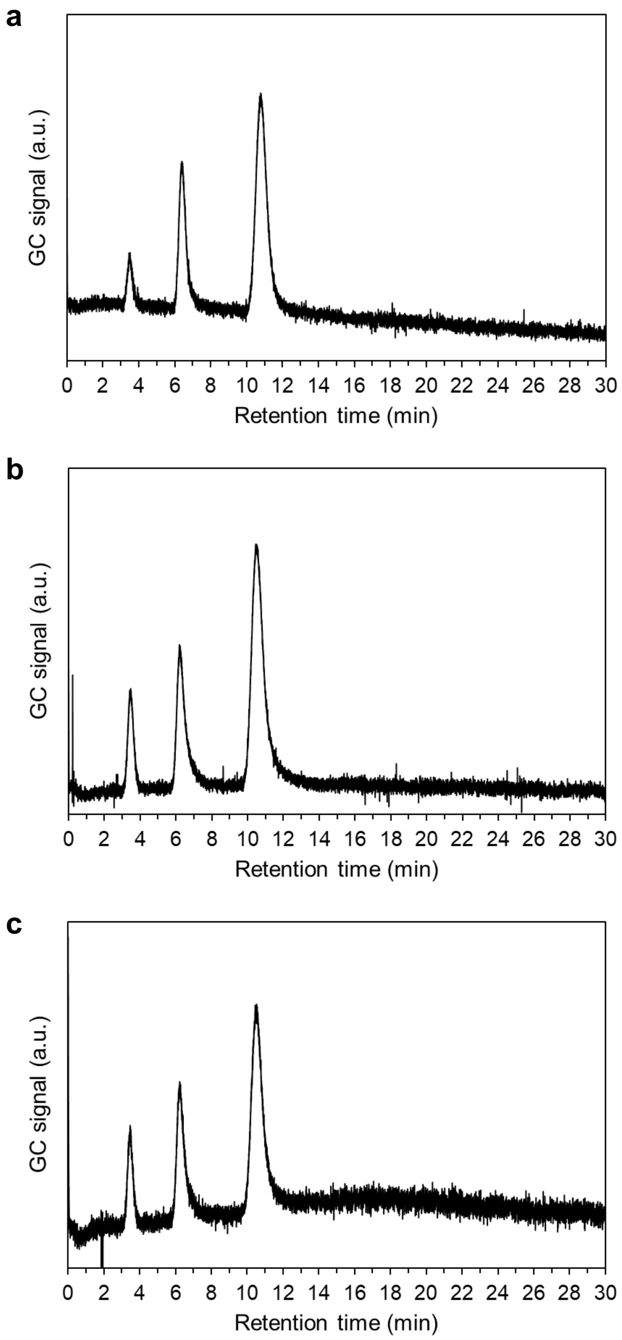

Supplementary Fig. 15 | Detection of non-hydrocarbon products using GC-TCD. (a–c) Extended retention time-range GC-TCD chromatogram obtained at the end of a 10 h long Au-photocatalyzed CO_2 reduction reaction with 5 mol% EMIM-BF_4 and CW irradiation of 532 nm light (1 W cm^{-2}). Three identical trials were performed, the results from which are shown in the three panels above. The GC peaks were assigned to H_2, O_2, and N_2 by comparison with the reference chromatogram in Supplementary Fig. 13. No peaks associated with CO or other gaseous products were observed in the extended chromatograms.

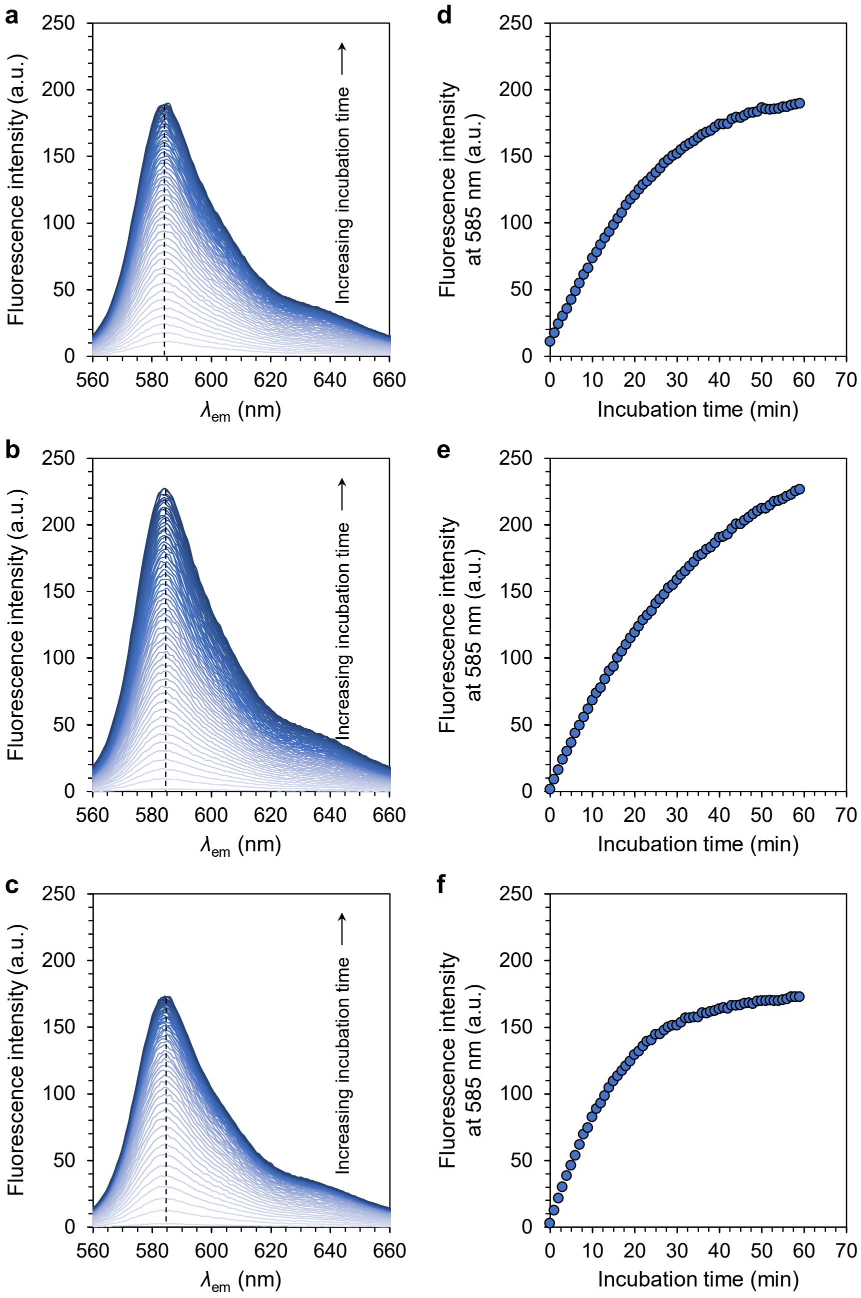

Supplementary Fig. 16 | Detection of H_2O_2 by a fluorogenic test involving the oxidation of amplex red to resorufin catalyzed by HRP. (a–c) Time-series of fluorescence emission spectra of an amplex red + HRP reagent solution subject to 100 μL of a post-reaction mixture obtained from 10 h of a Au NP-photocatalyzed CO_2 reduction reaction with 5 mol% EMIM-BF_4 and CW irradiation of 532 nm light (1 W cm^{-2}). The fluorescence spectra were recorded every minute for 1 h with an excitation wavelength of 550 nm. Three identical trials were performed, the results from which are shown one per row. The spectra show a fluorescence band centered around 585 nm, characteristic of resorufin. As shown by the (d–f) time-profiles of the fluorescence intensity at 585 nm, the intensity of this band increases with increasing incubation time with gradual tapering off over 1 h. This trend indicates the gradual oxidation of amplex red by H_2O_2 to fluorescent resorufin, confirming the presence of H_2O_2 in the post-reaction mixture.

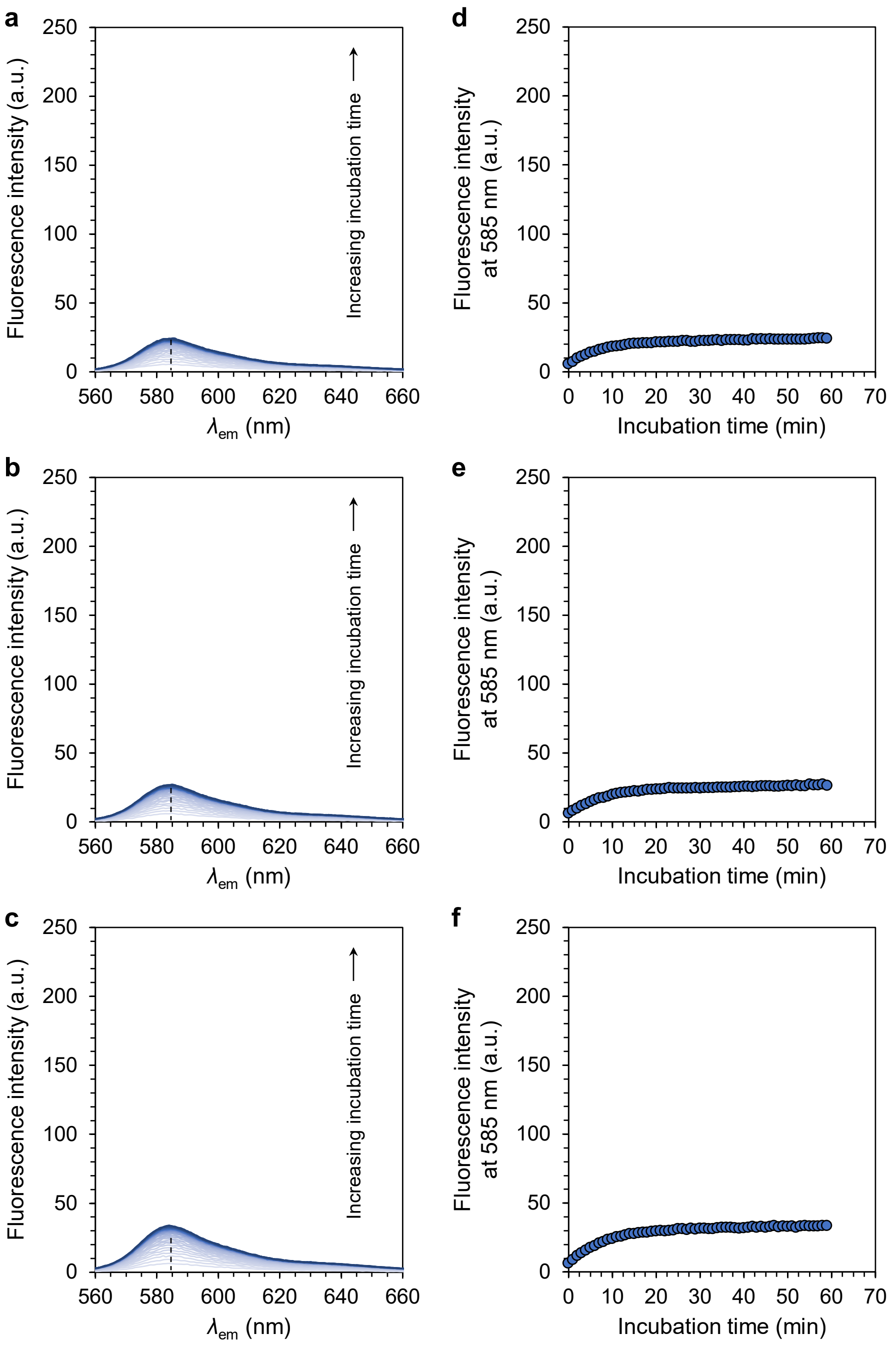

Supplementary Fig. 17 | Control test of the reagent consisting of amplex red and HRP. (a–c) Time-series of fluorescence emission spectra of an amplex red + HRP reagent solution subject to 100 µL of a reaction mixture prior to the photoreaction. The fluorescence spectra were recorded every minute at the excitation wavelength of 550 nm. Three identical trials were performed, the results from which are shown one per row. The spectra show a relatively weak fluorescence band, as compared to the spectra in Supplementary Fig. 16a–c. As shown by the (d–f) time-profiles of the fluorescence intensity at 585 nm, the intensity of this band increases for about 10–15 min, reaching saturation to a marginal value that corresponds to a background fluorescence of the reagent mixture. To allow appropriate comparison, all the plots have the same y-axis scale as the plots in Supplementary Fig. 16.

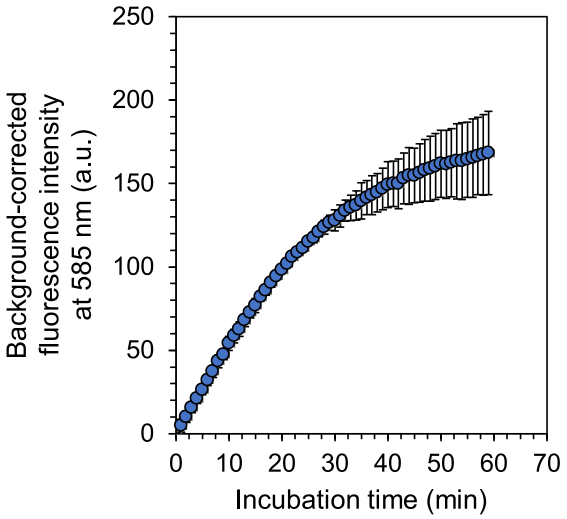

Supplementary Fig. 18 | Time-profile of the background-corrected fluorescence intensity at 585 nm in the fluorogenic test of the post-reaction mixture. The profile indicates an increase in the concentration of resorufin with increasing incubation time, approaching saturation over the 1 h period. Essentially, over the 1 h period, the amplex red is oxidized to fluorescent resorufin by H_2O_2 in the post-reaction mixture. Thus, the fluorogenic test indicates the generation of H_2O_2 in the photoreaction. For background correction, the fluorescence intensity measured in the fluorogenic test of the post-reaction mixture (Supplementary Fig. 16) was subtracted time-point-by-time-point from the fluorescence intensity measured in the control test of the pre-reaction mixture (Supplementary Fig. 17). The resulting background-corrected intensity was averaged across the three trials and plotted as a function of the incubation time. The error bar represents the standard deviation in the background corrected intensity across the three trials. To allow appropriate comparison, this plot has the same y-axis scale as the plots in Supplementary Figs. 16 and 17.

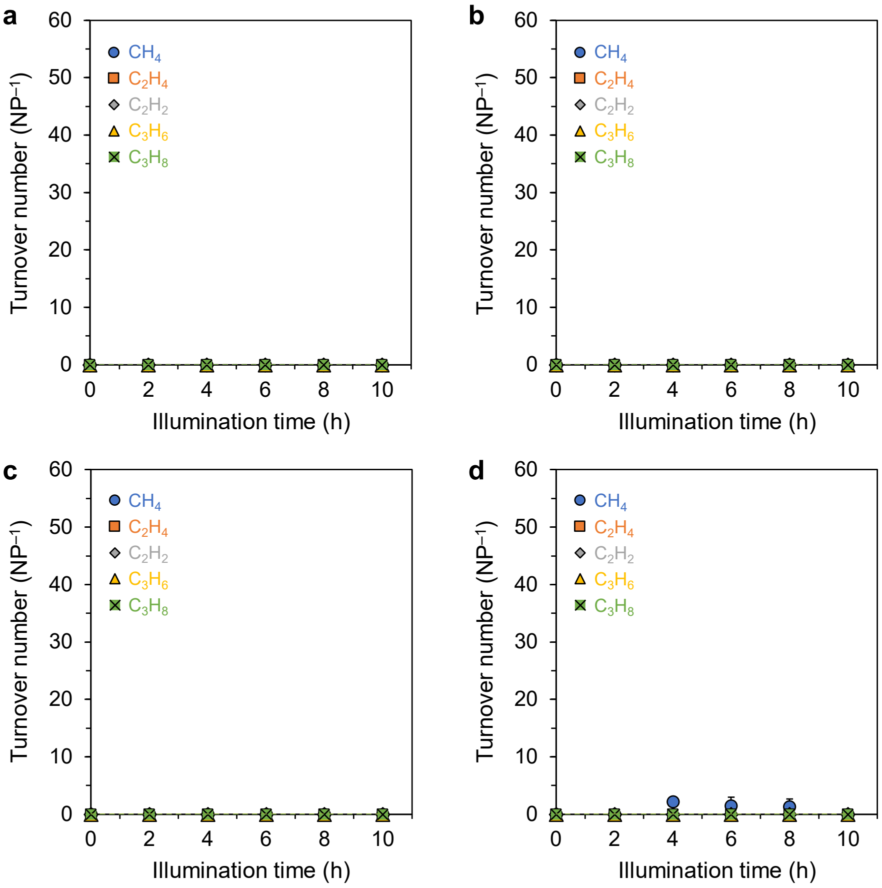

Supplementary Fig. 19 | Results of control studies. (a) Time course of product turnover in a photoreaction without Au NPs in 5 mol% EMIM-BF_4 solution saturated with CO_2 under 1 W cm^{-2}, 532 nm laser excitation. (b) Time course of product turnover in a dark reaction conducted at 50 °C with Au NPs in 5 mol% EMIM-BF_4 solution saturated with CO_2. The reaction temperature for this dark reaction is close to the bulk steady-state temperature (~48 °C) of the reaction mixture measured in photoreaction tests under 1 W cm^{-2}, 532 nm laser excitation. (c) Time course of product turnover in a photoreaction with Au NPs in 5 mol% EMIM-BF_4 solution under Ar atmosphere and 1 W cm^{-2}, 532 nm laser excitation. (d) Time course of product turnover in a photoreaction with Au NPs in CO_2-saturated DI water of pH 2.93 and 1 W cm^{-2}, 532 nm laser excitation. The solution pH was adjusted using 0.1 M HNO_3 to be similar to the measured pH (~2.95) of the reaction mixture with 5 mol% EMIM-BF_4. Negligible product generation was observed in these control experiments. Each data point is an average of measurements from two identical trials and the error bar represents the standard deviation of the measurements. All plots here are presented on the same scale as the plots in Supplementary Fig. 1.

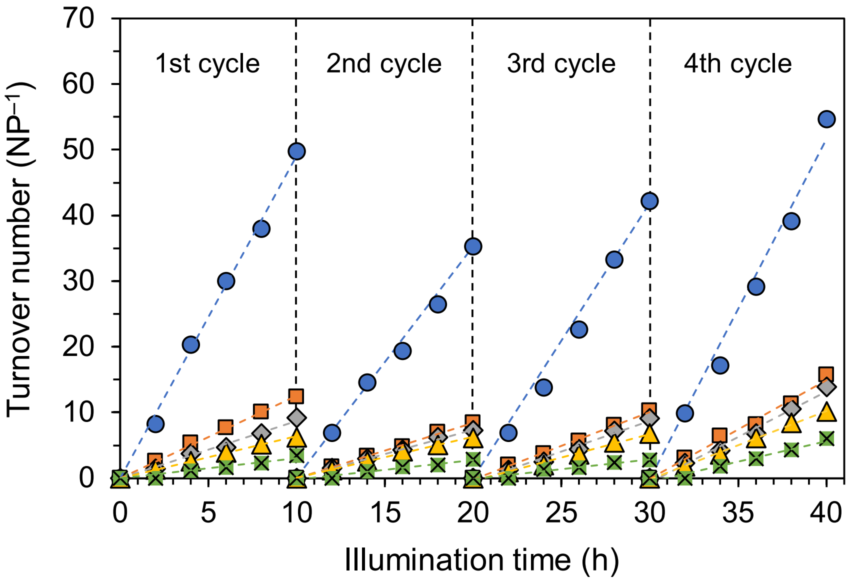

product	TOF (NP^{-1} h^{-1})			
	1st cycle	2nd cycle	3rd cycle	4th cycle
● CH_4	4.91	3.43	4.06	5.12
■ C_2H_4	1.26	0.85	0.99	1.49
◆ C_2H_2	0.87	0.74	0.85	1.30
▲ C_3H_6	0.63	0.63	0.65	1.02
☒ C_3H_8	0.30	0.27	0.28	0.54

Supplementary Fig. 20 | Time course of product turnover over four sequential cycles of Au-photocatalyzed CO_2 reduction reaction performed with 5 mol% EMIM-BF_4 solution under CW irradiation of 532 nm light (1 W cm^{-2}). After each 10 h cycle (demarcated by vertical lines), the reaction mixture was bubbled with Ar gas to remove gaseous products from the previous cycle. The mixture was then saturated with CO_2 by bubbling it with CO_2 for 20 min. The next photoreaction cycle was then conducted. All cycles were performed under identical conditions. The TON for each product (see color legend in the table below) is plotted as a function of illumination time. Each plot is fit to a linear plot (dashed line), the slope of which yields the TOF for that product. The measured TOF for each product is listed in the table for the four photoreaction cycles.

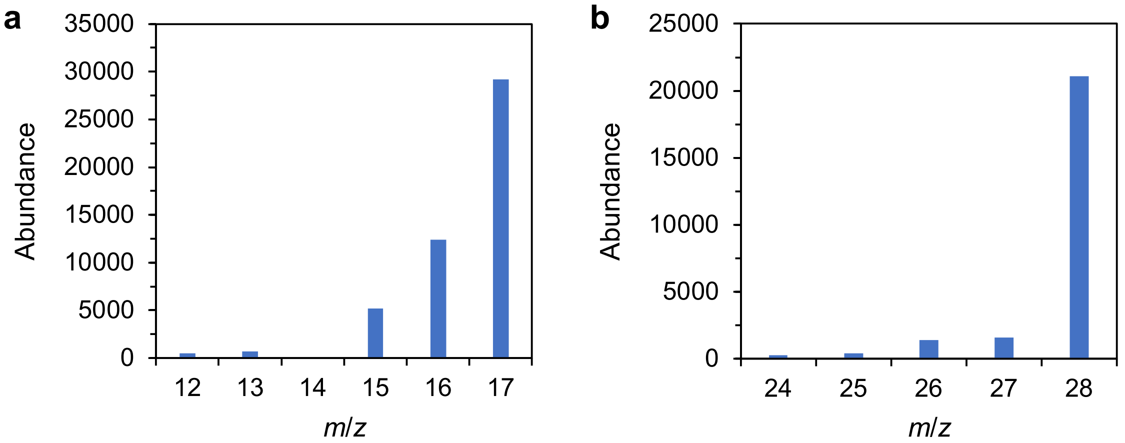

Supplementary Fig. 21 | Experimentally measured mass spectra at retention times of (a) 4.6 min and (b) 8.2 min of the TIC (Fig. 2a). The TIC and mass spectra were obtained by GC-MS analysis of the headspace gas collected after 240 h of a Au NP-photocatalyzed $^{13}CO_2$ reduction reaction with 5 mol% EMIM-BF_4 and CW irradiation of 532 nm light (1 W cm^{-2}). For the mass spectrum shown in (a), fragments at *m*/*z* = 14 and 18 were suppressed to remove the mass peaks contributed by N_2 and moisture, respectively. The fragment at *m*/*z* = 28 in (b) has relatively high abundance as compared to that of the reference fragmentation pattern (Fig. 2c) due to the contribution of N_2 from the atmosphere (Fig. 2a).

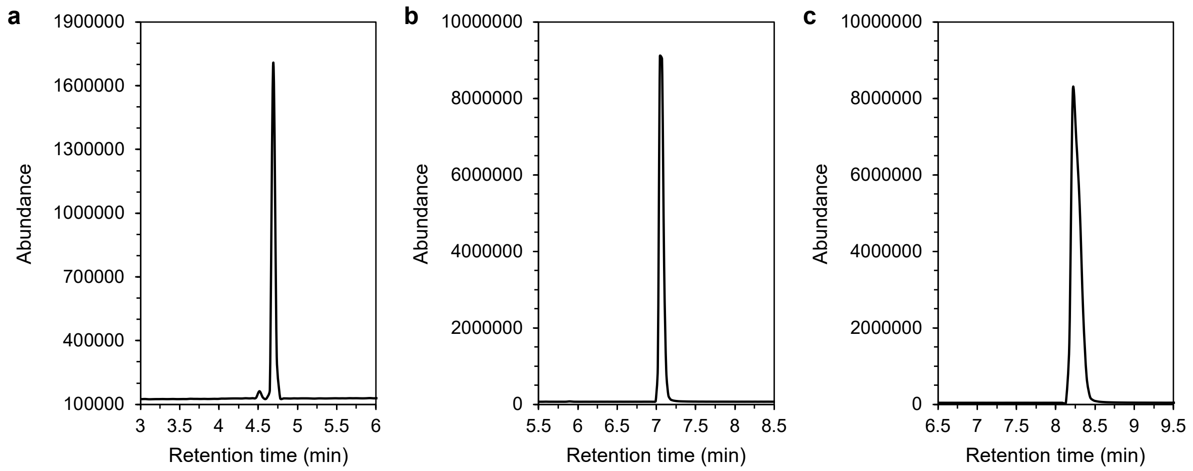

Supplementary Fig. 22 | Total ion chromatograms (TICs) of (a) $^{12}CH_4$, (b) $^{12}C_2H_4$, and (c) $^{12}C_2H_2$ reference gases. TIC peaks of $^{12}CH_4$, $^{12}C_2H_4$, and $^{12}C_2H_2$ are centered at retention times of 4.6 min, 7.1 min, and 8.2 min, respectively. Peaks observed in the TIC in Fig. 2a were assigned to $^{13}CH_4$ and $^{13}C_2H_2$ by comparison with these reference TICs and by the characteristic mass fragmentation patterns of these isotopologues. From the peak position of $^{12}C_2H_4$ in the TIC, we deduce that the TIC peak expected for $^{13}C_2H_4$ is overshadowed by the broad, intense peak of $^{13}CO_2$ in the TIC (Fig. 2a).

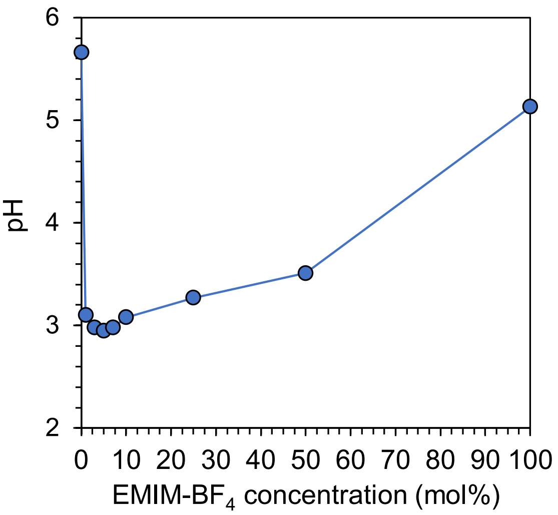

Supplementary Fig. 23 | The measured pH of reaction mixtures with different concentrations of EMIM-BF_4. The pH was measured using a pH meter (Mettler-Toledo AG 8603) in air at room temperature prior to bubbling of CO_2 gas, however CO_2 bubbling does not significantly change the pH, except in the 0 mol% case.

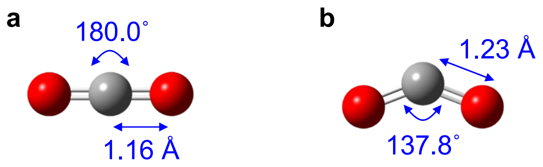

Supplementary Fig. 24 | DFT-optimized geometries of (a) CO_2 and (b) $CO_2^{\bullet-}$ where C and O atoms are depicted by gray and red spheres, respectively. Bond angles and bond lengths are indicated.

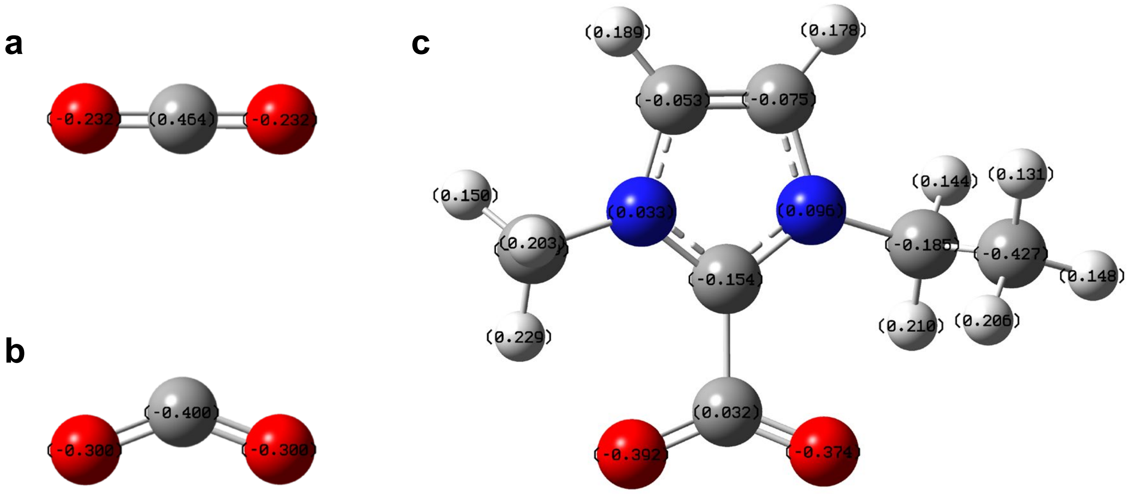

Supplementary Fig. 25 | Mulliken charges on each atom of (a) CO_2, (b) $CO_2^{\bullet-}$, and (c) [EMIM*-CO_2] where C, O, H, and N atoms are depicted by gray, red, white, and blue spheres, respectively. The net charge on the CO_2 moiety in [EMIM*-CO_2] is found to be 0.032-0.392-0.374 = -0.734.

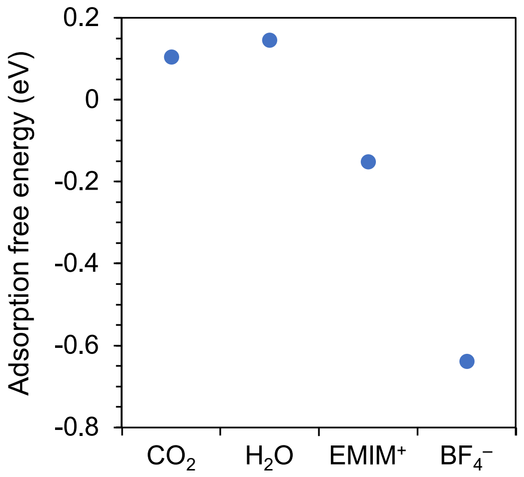

Supplementary Fig. 26 | Free energy of adsorption, ΔG_{ads}, of CO_2, H_2O, $EMIM^+$, and BF_4^- on a Au(111) surface. While absolute values of ΔG_{ads} estimated by DFT may not be representative due to the difficulty of estimating adsorption entropies accurately[1] and other limitations associated with the approximate model of the transition metal surface, these ΔG_{ads} values provide a relative measure of the adsorption strength of different species. The adsorption strength is predicted to increase in the order of H_2O, CO_2, $EMIM^+$, and BF_4^-.

Supplementary Notes

Supplementary Note 1 | Half-reaction potentials

Supplementary Equations (1)–(5) list the standard reduction half-reaction potentials, E°, for the conversion of CO_2 to the C_1−C_3 hydrocarbons. For comparison, E° is also provided for the competing half-reaction involving the reduction of H^+ (Supplementary Equation (6)).

$$CO_2 + 8H^+ + 8e^- \rightarrow CH_4 + 2H_2O \qquad E^\circ = 0.17\ V \qquad (1)$$

$$2CO_2 + 10H^+ + 10e^- \rightarrow C_2H_2 + 4H_2O \qquad E^\circ = -0.05\ V \qquad (2)$$

$$2CO_2 + 12H^+ + 12e^- \rightarrow C_2H_4 + 4H_2O \qquad E^\circ = 0.08\ V \qquad (3)$$

$$3CO_2 + 18H^+ + 18e^- \rightarrow C_3H_6 + 6H_2O \qquad E^\circ = 0.10\ V \qquad (4)$$

$$3CO_2 + 20H^+ + 20e^- \rightarrow C_3H_8 + 6H_2O \qquad E^\circ = 0.14\ V \qquad (5)$$

$$2H^+ + 2e^- \rightarrow H_2 \qquad E^\circ = 0.00\ V \qquad (6)$$

The E° values were determined from the standard Gibbs free energy of formation, ΔG_f° of the molecules involved in each reaction[2] and are provided with reference to the standard hydrogen electrode (SHE) and at a pH of 0. The Fermi level, E_F, of Au is at a potential of 0.66 V vs SHE at a pH of 0. The photon energy, hv, used for electronic excitation is 2.33 eV (λ = 532 nm). Electrons at the Fermi surface can be photoexcited to a potential as high as 0.66 V - 2.33 V = -1.67 V, which is sufficiently more negative than the E° for the above-listed reactions. In other words, these electrons are sufficiently energetic for driving the above-listed reduction half-reactions under standard conditions. Aside from the thermodynamic feasibility, the kinetics of hot electron transfer from the Au NP to CO_2 will depend on the free energy barrier. In the presence of EMIM-BF_4, the free energy barrier or overpotential is expected to be considerably lower, as per our estimates (Fig. 3d) and previous work on electrochemical CO_2RR[3–7].

Note that the accompanying photogenerated hole needs to be energetic enough for the oxidation of H_2O. The photon energy of 2.33 eV is larger than the ~1.8 eV threshold for inter-band excitation, which results in an excited hole in the d-band of the Au and an excited electron in the sp band[8]. For instance, when an excited electron is generated in the sp band with a potential of 0.17 V, just energetic enough for the methane generation half-reaction (Supplementary Equation (1)), the accompanying hole can be at a potential as positive as 0.17 V + 2.33 V = 2.50 V. This potential lies just at the edge of the d-band but it is more positive than the E° for the reaction:

$$H_2O_2 + 2H^+ + 2e^- \rightarrow 2H_2O \qquad E^\circ = 1.83\ V \qquad (7)$$

Supplementary Methods

Synthesis and characterization of Au nanoparticles (NPs)

The synthesis of 12 nm Au NPs was carried out as described previously[9]. As per this published procedure, a 50 mL aqueous solution of 0.5 mM $HAuCl_4 \cdot 3H_2O$ (≥ 49.0%, Sigma-Aldrich) was heated in a flask to 80 °C under constant stirring. When the solution temperature became stable, a 1.5 mL aqueous solution of 0.17 M trisodium citrate (99%, Alfar Aesar) was rapidly added to the flask. The ensuing reaction was continued at 80 °C for 20 min, during which the reaction mixture turned red in color. The resulting Au colloid was cooled down to room temperature. Then, 500 mg of polyvinylpyrrolidone (PVP, MW = 40,000, Amresco) was added to the colloid. The colloid was then sonicated for 1 h. Unreacted precursors and salts left in solution were removed by centrifugation (Eppendorf 5804) of the colloid at 11,000 rpm (15,557 g) for 30 min. This process was carried out thrice. The precipitate of each step was washed with deionized (DI) water. The final precipitate was dispersed in DI water to yield a stock colloid of Au NPs. The Au NPs have a pseudospherical shape and an average diameter of ~12 nm as characterized previously[9] using high resolution transmission electron microscopy (HRTEM) imaging. Extinction spectra of the Au NP colloids were recorded on an UV–Vis spectrophotometer (Shimadzu UV-3600). The UV–vis extinction spectrum exhibits a characteristic localized surface plasmon resonance (LSPR) band centered around 520 nm. The molar concentration, *C*, of Au NPs in the stock colloid was estimated using Beer-Lambert's law:

$$C = \frac{A}{\varepsilon b} \tag{8}$$

where *A* is the absorbance at the peak wavelength of the LSPR band, *ε* is the extinction coefficient at this peak wavelength for ~12 nm diameter Au NPs (1.891×10^{8} M^{-1} cm^{-1})[10], and *b* is the path length of the solution in the UV–vis spectrophotometry cuvette (1 cm). The molar concentration, *C*, was used to determine the number of moles of Au NPs in a given volume of the stock colloid.

Photocatalytic CO_2 reduction reaction

Photocatalytic reactions were performed in an air-tight Pyrex cell of 10 mL volume. A known concentration (0, 1, 3, 5, 7, 10, 25, 50, or 100 mol%) of the ionic liquid (IL), 1-ethyl-3-methylimidazolium tetrafluoroborate ($EMIM\text{-}BF_4$, Sigma-Aldrich), was prepared in DI water. Prior to use of the IL, moisture and oxygen dissolved in the IL were removed. Dehydration

and degassing were achieved by applying a vacuum (< 100 mTorr) to a flask containing the IL heated at 100 °C for 4 h and then refilling the flask with Ar (> 99.999%) gas. Three mL of the aqueous IL solution were transferred to the Pyrex reaction cell.

A few mL of Au colloid containing a net ~3.175×10^{-11} mol of NPs (i.e., 1.912×10^{13} NPs), was drop-coated on a 1.5 cm × 1.5 cm cotton cloth substrate (Texwipe TX 306) that was free of chemical additives. With the aim of cleaning off surface ligands and potential contaminants, the NP-coated substrate was washed with 60 °C DI water and then rinsed with room temperature DI water three times, followed by drying overnight in an oven at 70 °C. The resulting substrate was immersed in the IL solution contained in the Pyrex cell. The resulting solution was saturated with CO_2 by flowing CO_2 gas (> 99.999%) through the solution at a rate of 10 mL min^{-1} for 20 min in the dark. Then, the solution was stirred for 30 min to 1 h to facilitate adsorption-desorption equilibrium.

The photoreaction was conducted by irradiation of the above-prepared reaction mixture by a 532 nm wavelength beam from a continuous-wave (CW) solid-state diode laser (UltraLasers CST-H-532-1500). The incident laser power was 1 W measured by an optical power meter (ThorLabs PM100D) connected to a thermal power sensor (ThorLabs S310C). The incident beam was directed onto the substrate-supported Au NP film. The beam spot had an area of ~1 cm^2 at the substrate. The laser intensity was 1 W cm^{-2}. Gaseous products in the photoreaction collected in the headspace of the reactor. The products in the headspace were analyzed at periodic intervals using an Agilent Technology 6850 series gas chromatograph (GC) equipped with a flame ionization detector (FID). For measuring production of H_2, CO, and O_2, an Agilent Technology 7820A GC equipped with a thermal conductivity detector (TCD) was employed.

Control studies were also performed: one without CO_2, one without Au NPs, and another without light. These studies were performed in a 5 mol% EMIM-BF_4 solution, the IL concentration at which the activity was found to be the highest in this work. For the control study without CO_2, the reaction mixture was instead bubbled with Ar gas for 30 min to 1 h, but all other conditions were kept the same as the photoreaction tests. For the control study without Au NPs, a bare substrate (with no Au NPs) was employed, whereas all other conditions, including the presence of a CO_2 saturated solution, were kept the same as the photoreaction tests. For the control study without light excitation, all conditions were kept the same as the photoreaction tests, however the reaction mixture was maintained in the dark at

50 °C, which nearly corresponds to the steady-state bulk solution temperature (~48 °C) of the reaction mixture in the photoreaction tests under 1 W cm^{-2}, 532 nm laser excitation.

Data analysis

Photoreaction activity was determined by analysis of chromatograms obtained by GC-FID measurement at specific time intervals in the photoreaction, as described in the past[9]. GC peaks detected in chromatograms were first assigned by comparison with reference chromatograms (see Supplementary Fig. 2 for hydrocarbon products and Supplementary Fig. 13 for non-hydrocarbon products). The chromatogram measured at each time interval was subtracted from the initial chromatogram to remove the GC peak contribution of the trace CH_4 pre-present in the CO_2 gas as an impurity. In control studies, where there is little to no product formation, the latter subtraction procedure can yield a negative GC peak for CH_4 due to a systematic measurement error. In such cases, the amount of CH_4 produced was regarded as zero.

From each chromatogram, areas of GC peaks of specific products were determined. The integrated GC peak area was converted into a molar amount of the product by taking into account the calibration constant determined from reference chromatograms for the product. This molar amount of the product in the injection volume was multiplied by the ratio of the headspace volume to the injection volume to obtain the moles of product in the headspace. A correction was also performed for the cumulative loss of product from the headspace due to the prior injections. The cumulative molar amount of product generated up to a specific illumination time was divided by the moles of Au NPs in the reaction mixture to obtain a turnover number (TON), i.e., the number of product molecules generated per Au NP. For this TON estimate, we used the moles of Au NPs present in the colloid, which was drop-casted to form the substrate-supported photocatalyst film. However, it is plausible that a fraction of the Au NPs were lost during the deposition process. Secondly, not all the Au NPs deposited on the substrate were located within the irradiation zone of the laser beam. Therefore, the TON we estimated represents a lower limit and the actual TON is likely higher. The slope of a linear fit to the plot of the TON vs. illumination time yielded the turnover frequency (TOF). Product selectivity was determined by dividing the TOF of a specific product generated in the photoreaction by the sum of TOFs of all products generated in the photoreaction.

H_2 and O_2 produced in the reaction were quantified from chromatograms measured using GC-TCD. The measured GC peak areas of H_2, O_2, and N_2 were converted to molar amounts

of these gases by multiplying by their respective calibration constants. In the case of O_2, it was necessary to account for O_2 that results from air contamination and does not originate from photoproduction. From the molar amount of N_2 measured and the known molar ratio of O_2/N_2 in the atmosphere, we determined the molar amount of O_2 that simply originated from air leftover in the reactor from the reaction mixture preparation or from any air leakage that may have occurred over the course of the photoreaction or during injection of the headspace gas into the GC. The molar amount of O_2 determined to be from air (and not photoproduction) was subtracted out from the total molar amount of O_2 determined from the GC-TCD chromatogram. However, this correction does not fully remove the contribution from O_2 dissolved in the reaction mixture. This limitation arises because O_2 has a two-fold higher solubility in water compared to N_2. This dissolved O_2 amount greatly overwhelms any amount of O_2 photoproduced in the reaction, the generation rate of which is estimated to be on the order of 100 NP^{-1} h^{-1} as per the following sequence of reactions (Supplementary Equations (9)−(14)):

$$CO_2 + 4H_2O \rightarrow CH_4 + 2H_2 + 3O_2 \quad (9)$$

$$2CO_2 + 4H_2O \rightarrow C_2H_4 + 2H_2 + 4O_2 \quad (10)$$

$$2CO_2 + 2H_2O \rightarrow C_2H_2 + H_2 + 3O_2 \quad (11)$$

$$3CO_2 + 4H_2O \rightarrow C_3H_6 + H_2 + 5O_2 \quad (12)$$

$$3CO_2 + 6H_2O \rightarrow C_3H_8 + 2H_2 + 6O_2 \quad (13)$$

$$2H_2O \rightarrow 2H_2 + O_2 \quad (14)$$

Therefore, it was difficult to determine if O_2 was generated as an oxidation product of the reaction.

Hydrogen peroxide (H_2O_2) detection

H_2O_2 is another possible oxidation product as per the following sequence of reactions (Supplementary Equations (15)−(20)):

$$CO_2 + 6H_2O \rightarrow CH_4 + 4H_2O_2 \quad (15)$$

$$2CO_2 + 8H_2O \rightarrow C_2H_4 + 6H_2O_2 \quad (16)$$

$$2CO_2 + 6H_2O \rightarrow C_2H_2 + 5H_2O_2 \quad (17)$$

$$3CO_2 + 12H_2O \rightarrow C_3H_6 + 9H_2O_2 \quad (18)$$

$$3CO_2 + 14H_2O \rightarrow C_3H_8 + 10H_2O_2 \quad (19)$$

$$2H_2O \rightarrow H_2 + H_2O_2 \quad (20)$$

In fact, the two-hole oxidation of water to H_2O_2 ($2H_2O \rightarrow H_2O_2 + 2H^+ + 2e^-$) is the kinetically favored oxidation half-reaction as compared to the four-hole-oxidation of water to O_2 ($2H_2O \rightarrow O_2 + 4H^+ + 4e^-$). To detect H_2O_2, we performed a well-known fluorogenic test[11] using a reagent kit (Invitrogen) consisting of the dye amplex red and the enzyme horseradish peroxidase (HRP). The non-fluorescent amplex red reacts with H_2O_2 in a 1:1 stoichiometry, catalyzed by HRP, to form highly fluorescent resorufin. Resorufin has an excitation maximum, λ_{ex}, at ~570 nm, a fluorescence emission maximum, λ_{em}, at ~585 nm, and a molar extinction coefficient of ε = 58,000 ± 5,000 cm^{-1} M^{-1} (ref. [11]). For the detection of H_2O_2 in our post-reaction mixture, a reagent solution was prepared in a gas-tight cuvette by mixing the following: 10 μL of 100 μM amplex red stock solution in dimethyl sulfoxide (DMSO), 10 μL of 1 U/mL HRP stock solution in 50 mM phosphate buffer (pH 7.4), and 2.88 mL of 50 mM phosphate buffer (pH 7.4). For reference, 1 unit (U) is defined as the amount of enzyme that forms 1.0 mg purpurogallin from pyrogallol in 20 s at a pH of 6.0 and 20 °C. The reaction mixture that was subject to the H_2O_2 analysis was obtained by a photoreaction conducted (as described in the 'Photocatalytic CO_2 reduction reaction' section) in 5 mol% EMIM-BF_4 for 10 h. A 100 μL sample from the post-reaction mixture was injected using a micro-syringe into 2.9 mL of the amplex red and HRP reagent solution. The fluorescence spectrum of the resulting mixture was measured in the range of 560–660 nm with an excitation wavelength of 550 nm at room temperature every 1 min for 1 h. For testing reproducibility, three independent trials were conducted in the same manner, the results from which are shown in Supplementary Fig. 16. The fluorescence spectrum showed the characteristic fluorescence band of resorufin with an λ_{em} around 585 nm. The band grows stronger with increasing incubation time gradually tapering off over 1 h. This trend indicates the gradual conversion of amplex red to resorufin by H_2O_2 in the mixture. A control test was also conducted (Supplementary Fig. 17) in which a 100 μL sample taken from the reaction mixture at t = 0 (prior to photoreaction) was subject to the fluorogenic test exactly as described for the post-reaction mixture. Three independent trials of this control test were performed, the results of which are shown in Supplementary Fig. 17. In these control tests, we measured a relatively weak fluorescence emission, which saturates in intensity after 10–

15 min and corresponds to a background fluorescence of the reagent mixture.

Isotope labeling studies

Photoreaction studies with the $^{13}CO_2$ gas (99 atom% ^{13}C, Aldrich) were conducted in 5 mol% EMIM-BF_4 solution. All other conditions and procedures were the same as those for the photoreactions involving regular CO_2 gas. Following 240 h of photoreaction, the gaseous products in the reactor headspace were subject to gas chromatography-mass spectrometry (GC-MS) analysis. Total ion chromatogram and mass spectra were acquired on a HP 5890 series II GC system equipped with a HP 5971A mass selective detector. The mass spectrometer was operated in electron-impact ionization mode. Mass peaks at m/z = 14 and 18 were suppressed to exclude the contribution from ambient N_2 and moisture, but the mass peak at m/z = 28 was included for analysis of $^{13}C_2H_2$ produced in the photoreaction. The fragmentation patterns of the products measured by mass spectrometry were compared with those deposited in the National Institute of Standards and Technology (NIST) Chemistry WebBook.

Computational study

Density functional theory (DFT) calculations were performed with the Gaussian 09 program. All species studied were geometry-optimized using the B3LYP functional and the 6-311++G(d,p) basis set for C, H, O, and N atoms. The geometry of the [EMIM*-CO_2] was based on a previously reported structure[12–16], wherein an $EMIM^+$ cation undergoes H^+ loss to form EMIM*, which is followed by complexation with CO_2. The geometry optimized [EMIM*-CO_2] complex exhibited a covalent linkage between the C atom of the CO_2 and the C_2 atom of the imidazole ring. A $CO_2^{\bullet-}$ anion was specified by assigning a charge of -1 to a CO_2 molecule and geometry-optimizing the resulting radical anion. Mulliken charge distributions were obtained as part of the output of the geometry-optimization procedure and viewed in GaussView, saved images from which are presented for CO_2, $CO_2^{\bullet-}$, and [EMIM*-CO_2] in Supplementary Fig. 25. The energy of intermolecular interaction, $E_{\text{m-m}}$, was calculated as:

$$E_{\text{m-m}} = E(\text{combined molecule}) - E(\text{molecule 1}) - E(\text{molecule 2}) \quad (21)$$

where E is the total energy of the geometry-optimized species indicated in parentheses. Free energies, G, given by the sum of electronic and thermal free energies:

$$G = \varepsilon_0 + G_{\text{corr}} \quad (22)$$

were determined for the geometry-optimized species CO_2 and [EMIM*-CO_2] and their respective 1e^- adducts: $CO_2^{\bullet-}$ and [EMIM*-CO_2]$^{\bullet-}$. The adducts were generated by assigning a global charge of -1 to the geometry-optimized species, which was followed by geometry-optimization in the charged state. Free energies were also determined for [EMIM-CO_2]$^{\bullet}$, which is the geometry-optimized complex formed by the 1e^- adduct of CO_2, $CO_2^{\bullet-}$, and $EMIM^+$. The free energy of adsorption, ΔG_{ads}, was determined for CO_2, H_2O, $EMIM^+$, and BF_4^- on Au from DFT-calculated free energies as:

$$\Delta G_{ads} = G(\text{molecule/Au}) - G(\text{molecule}) - G(\text{Au}) \quad (23)$$

where G is the free energy of the geometry-optimized species indicated in parentheses. For this calculation, a two-layer Au slab consisting of 15 atoms was modeled using the face-centered cubic (fcc) structure (*Fm-3m* (2 2 5), a = 4.0786 Å) with the (111) surface exposed. The (111) facets are experimentally determined to be the most prevalent surface facet on Au NPs. Geometries of the constructed Au slab, adsorbates by themselves, and adsorbate/Au slab complexes were optimized using the B3LYP functional, the LANL2DZ basis set for outer shell electrons of Au atom, the LANL2 effective core potential (ECP) for core electrons of Au atom, and the 6-311++G(d,p) basis set for C, H, O, and N atoms. In every geometry optimization procedure, the upper layer of the Au slab was frozen to maintain the surface facet, and the other lower layer was allowed to relax.

Supplementary References

1. Campbell, C. T. & Sellers, J. R. V. The entropies of adsorbed molecules. *J. Am. Chem. Soc.* **134**, 18109−18115 (2012).

2. Dean, J. A. *Lange's Handbook of Chemistry* 15th edn (MGraw-Hill Inc., 1998).

3. Rosen, B. A. *et al.* Ionic liquid–mediated selective conversion of CO_2 to CO at low overpotentials. *Science* **334**, 643–644 (2011).

4. García Rey, N. & Dlott, D. D. Structural transition in an ionic liquid controls CO_2 electrochemical reduction. *J. Phys. Chem. C* **119**, 20892–20899 (2015).

5. Asadi, M. *et al.* Nanostructured transition metal dichalcogenide electrocatalysts for CO_2 reduction in ionic liquid. *Science* **353**, 467–470 (2016).

6. Rosen, B. A. *et al.* In situ spectroscopic examination of a low overpotential pathway for carbon dioxide conversion to carbon monoxide. *J. Phys. Chem. C* **116**, 15307–15312 (2012).

7. Wang, Y. *et al.* Activation of CO_2 by ionic liquid EMIM–BF_4 in the electrochemical system: a theoretical study. *Phys. Chem. Chem. Phys.* **17**, 23521–23531 (2015).

8. Ngoc, L. L. T., Wiedemair, J., Berg, A. Van Den & Carlen, E. T. Plasmon-modulated photoluminescence from gold nanostructures and its dependence on plasmon resonance, excitation energy, and band structure. *Opt. Express* **23**, 5547–5564 (2015).

9. Yu, S., Wilson, A. J., Heo, J. & Jain, P. K. Plasmonic control of multi-electron transfer and C-C coupling in visible-light-driven CO_2 reduction on Au nanoparticles. *Nano Lett.* **18**, 2189–2194 (2018).

10. Jain, P. K., Lee, K. S., El-Sayed, I. H. & El-Sayed, M. A. Calculated absorption and scattering properties of gold nanoparticles of different size, shape, and composition: applications in biological imaging and biomedicine. *J. Phys. Chem. B* **110**, 7238–7248 (2006).

11. Zhou, M., Diwu, Z., Panchuk-Voloshina, N. & Haugland, R. P. A stable nonfluorescent derivative of resorufin for the fluorometric determination of trace hydrogen peroxide: applications in detecting the activity of phagocyte NADPH oxidase and other oxidases. *Anal. Biochem.* **253**, 162–168 (1997).

12. Voutchkova, A. M., Feliz, M., Clot, E., Eisenstein, O. & Crabtree, R. H. Imidazolium carboxylates as versatile and selective N-heterocyclic carbene transfer agents: synthesis, mechanism, and applications. *J. Am. Chem. Soc.* **129**, 12834–12846 (2007).

13. Gurau, G. *et al.* Demonstration of chemisorption of carbon dioxide in 1,3-dialkylimidazolium acetate ionic liquids. *Angew. Chem. Int. Ed.* **50**, 12024–12026 (2011).

14. Fèvre, M. *et al.* Imidazol(in)ium hydrogen carbonates as a genuine source of N-heterocyclic carbenes (NHCs): applications to the facile preparation of NHC metal complexes and to NHC-organocatalyzed molecular and macromolecular syntheses. *J. Am. Chem. Soc.* **134**, 6776–6784 (2012).

15. Sun, L., Ramesha, G. K., Kamat, P. V & Brennecke, J. F. Switching the reaction course

of electrochemical CO_2 reduction with ionic liquids. *Langmuir* **30**, 6302–6308 (2014).

16. Lu, W. *et al.* Efficient photoelectrochemical reduction of carbon dioxide to formic acid: a functionalized ionic liquid as an absorbent and electrolyte. *Angew. Chem. Int. Ed.* **56**, 11851–11854 (2017).
